# Drought Tolerant Near Isogenic Lines of Pusa 44 Pyramided With *qDTY2.1* and *qDTY3.1*, Show Accelerated Recovery Response in a High Throughput Phenomics Based Phenotyping

**DOI:** 10.3389/fpls.2021.752730

**Published:** 2022-01-05

**Authors:** Priyanka Dwivedi, Naleeni Ramawat, Dhandapani Raju, Gaurav Dhawan, S. Gopala Krishnan, Viswanathan Chinnusamy, Prolay Kumar Bhowmick, K. K. Vinod, Madan Pal, Mariappan Nagarajan, Ranjith Kumar Ellur, Haritha Bollinedi, Ashok K. Singh

**Affiliations:** ^1^Division of Genetics, ICAR-Indian Agricultural Research Institute (ICAR-IARI), New Delhi, India; ^2^Amity Institute of Organic Agriculture, Amity University, Noida, India; ^3^Nanaji Deshmukh Plant Phenomics Centre, ICAR-IARI, New Delhi, India; ^4^Division of Plant Physiology, ICAR-IARI, New Delhi, India; ^5^Rice Breeding and Genetics Research Centre, ICAR-IARI, Aduthurai, India

**Keywords:** phenomics, controlled environment, image-based phenotyping, drought tolerance, rice

## Abstract

Reproductive stage drought stress (RSDS) is a major challenge in rice production worldwide. Cultivar development with drought tolerance has been slow due to the lack of precise high throughput phenotyping tools to quantify drought stress-induced effects. Most of the available techniques are based on destructive sampling and do not assess the progress of the plant’s response to drought. In this study, we have used state-of-the-art image-based phenotyping in a phenomics platform that offers a controlled environment, non-invasive phenotyping, high accuracy, speed, and continuity. In rice, several quantitative trait loci (QTLs) which govern grain yield under drought determine RSDS tolerance. Among these, *qDTY2.1* and *qDTY3.1* were used for marker-assisted breeding. A set of 35 near-isogenic lines (NILs), introgressed with these QTLs in the popular variety, Pusa 44 were used to assess the efficiency of image-based phenotyping for RSDS tolerance. NILs offered the most reliable contrast since they differed from Pusa 44 only for the QTLs. Four traits, namely, the projected shoot area (PSA), water use (WU), transpiration rate (TR), and red-green-blue (RGB) and near-infrared (NIR) values were used. Differential temporal responses could be seen under drought, but not under unstressed conditions. NILs showed significant level of RSDS tolerance as compared to Pusa 44. Among the traits, PSA showed strong association with yield (80%) as well as with two drought tolerances indices, stress susceptibility index (SSI) and tolerance index (TOL), establishing its ability in identifying the best drought tolerant NILs. The results revealed that the introgression of QTLs helped minimize the mean WU per unit of biomass per day, suggesting the potential role of these QTLs in improving WU-efficiency (WUE). We identified 11 NILs based on phenomics traits as well as performance under imposed drought in the field. The study emphasizes the use of phenomics traits as selection criteria for RSDS tolerance at an early stage, and is the first report of using phenomics parameters in RSDS selection in rice.

## Introduction

Abiotic stresses such as drought are detrimental to the growth, development, productivity, and grain quality in rice (Kumar et al., 2012; Raman et al., 2012), because more than 80% of its growth period is water-dependent. This renders drought as an extremely hazardous abiotic stress affecting rice production globally (Kumar, 2018). The threat due to drought becomes more pertinent in the wake of global climate change, owing to its frequent occurrence around the globe. In India, particularly in states such as Odisha, Chhattisgarh, eastern Uttar Pradesh, Bihar, and Jharkhand, recent episodes of severe drought had resulted in significant yield losses in rice (Kumar A. et al., 2014; Kumar S. et al., 2014). Some of these states suffered yield losses of up to 40%.^1^ Drought-related yield loss in rice during 2015–2016 alone is estimated to be 1.17 million tonnes (DACFW, 2017).

Drought is a complex phenomenon that combines interaction of various climatic, geographical, and edaphic factors resulting in deprivation of water for crop growth. Drought often occurs unpredicted after a failed rainfall and is commonly associated with high temperatures. Managing drought stress through agronomic practice after its onset is virtually unfeasible in rainfed ecosystems. Therefore, it becomes crucial to opt for preventive strategies against any drought occurrence during a crop cycle. Among the available options, developing rice varieties with inbuilt drought tolerance is the most strategic in terms of sustainability. Rice is naturally sensitive to drought stress, but the degree of damage depends on the affected crop stage, stress duration and intensity. Among the crop stages, drought incidence at the reproductive stage is most damaging in terms of economic returns. Therefore, reproductive stage drought stress (RSDS) tolerance is one of the most desirable attributes for cultivar development in rice. Drought tolerance is a complex quantitative trait with variable phenotypic impacts at different developmental stages (Oladosu et al., 2019). In the past, many quantitative trait loci (QTLs) have been mapped in rice, notably associated with RSDS tolerance and targeting grain yield under drought. However, utilizing these QTLs for the development of improved varieties requires a robust crop phenotyping strategy, which is efficient and time-saving (Tuberosa, 2012; Deery et al., 2014; Underwood et al., 2017).

Robust phenotyping has been implicated as a major impediment in evaluating breeding lines for drought tolerance in rice (Deery et al., 2016). The common methods in use often resulted in poor concordance of crop response between artificially managed and naturally occurring drought stress, leading to dubious conclusions (Courtois et al., 2000; Lafitte et al., 2006; Bernier et al., 2008; Venuprasad et al., 2009). In the traditional phenotyping methods, the poor precision is due to the limited use of actual crop response parameters, that are relatively simple, inaccurate, and intermittent. Besides, traditional drought phenotyping is laborious, time-consuming, hectic, economically ineffective, and plant destructive (Furbank and Tester, 2011; Chen et al., 2014). Notwithstanding, imposed drought treatments under natural field conditions had always been under the threat of unforeseen rains (Hoover et al., 2018). Although lately, facilities such as rain-out shelters have improved the screening system by preventing rain interference (Singh et al., 2016), they seldom offered improved fidelity in the response data. Rainout shelters are huge structures that require large land areas and are laborious to operate and maintain. Furthermore, the scaling up of rainout shelters to handle large populations becomes practically unfeasible after establishment.

Until the recent development of phenomics platforms, high throughput phenotyping has been seldom used in agricultural research (Lippman and Zamir, 2007). Assembled in an environment-controlled screen house, phenomics platforms provide image-based monitoring of crop ontogenesis on a continuous time scale. With its non-destructive phenotyping setup equipped to generate high-resolution data (Ubbens and Stavness, 2017), phenomics platforms help in screening for abiotic stress responses with the capabilities of handling large populations and automated data recording. The multidimensional imaging in the phenomics facilities uses visible, infrared (IR), near-IR (NIR), and hyperspectral bands, providing opportunities for temporal assessment of several physiological traits (Peñuelas and Filella, 1998; Tester and Langridge, 2010). For instance, the stomatal response to drought stress is an important trait for screening tolerance that is measured using thermal imaging and canopy temperature (Jones et al., 2009; Rischbeck et al., 2017). Likewise, several stress responses can be assessed through continuous crop monitoring.

Phenotyping in a phenomics platform is an automated process, in which each plant travels through a battery of imaging devices at pre-programmed intervals. During this process, several plant health parameters are monitored continuously to ensure ideal cultural environment for the test plants. Therefore, the phenotyping ensures accuracy and precision, while saving time and labor of handling large populations. The data generated are highly reliable than those generated from rainout shelters and field-based platforms. Contemporarily, the number of studies employing high throughput phenotyping is on the rise for studying quantitative inheritance of various traits in rice. Yang et al. (2015) could identify nine QTLs associated with leaf traits from a genome-wide association study (GWAS) involving 533 rice accessions. Similarly, Guo et al. (2018) employed 51 image-based traits to evaluate drought tolerance in 507 rice accessions. Dynamic quantification of RSDS response among 40 rice accessions grown under two cultural conditions, pot and field, using high throughput phenotyping, revealed that four traits, viz., greenness plant area ratio (GPAR), total plant area/bounding rectangle area ratio (TBR), total plant area/convex hull area ratio (TAR) and perimeter area ratio (PAR) were capable of differentiating drought resistant and susceptible genotypes (Duan et al., 2018). Kim et al. (2020) demonstrated the efficiency of red-green-blue (RGB) images in predicting plant area, color, and compactness; NIR images for assessing plant water content; IR images for assessing plant temperature and fluorescence; and gravimetric platform (DroughtSpotter^®^) for measurement of water use efficiency (WUE), plant water loss rate, and transpiration rate at various crop stages. They used the data to assess the drought response between a drought-tolerant rice mutant, *osphyb*, and its wild type (WT), by estimating photosynthetic efficiency. Although, high throughput phenotyping has been used to study drought and high-temperature responses in crops such as wheat (Shirdelmoghanloo et al., 2016), tomato (Danzi et al., 2019), and *Brassica* (Chen et al., 2019), the use of image-based screening for assessing stress tolerance in rice is still in its infancy. Therefore, detailed experimentations are needed for understanding the versatility of this method in breeding for tolerance to abiotic stresses such as drought.

Near isogenic lines (NILs) form an ideal set of experimental material for comparative phenotypic evaluation and assessment of the precision achieved in phenomics platforms. Unlike the mutants, NILs are easy to generate and provide relatively accurate genomic comparison. Moreover, several NILs can be simultaneously tested to generate comprehensive data suited for a reliable analysis. Marker-assisted backcross breeding (MABB) and genetic engineering are used to generate NILs in crops. MABB has been proved to be one of the best ways to incorporate target traits in cultivars (Dhawan et al., 2021; Singh et al., 2021). In recent years, several QTLs and meta-QTLs have been identified in rice which are associated with different levels of tolerance to drought. Further, several improved lines carrying these QTLs such as *qDTY1.1*, *qDTY2.1*, *qDTY2.2*, *qDTY3.1*, *qDTY4.1*, *qDTY3.2*, *qDTY9.1*, *qDTY10.1*, and *qDTY12.1* (Vinod et al., 2019; Dhawan et al., 2021; Dwivedi et al., 2021; Oo et al., 2021a) have been developed in rice.

In the present study, a set of 35 NILs developed in the background of the popular rice cultivar, Pusa 44 introgressed with two QTLs, *qDTY2.1* and *qDTY3.1* (Dwivedi et al., 2021) were evaluated in the field as well as in a phenomics facility, under induced RSDS. The objective was to identify the most promising NILs showing resilience under RSDS using different image-based parameters such as NIR and visual (VIS) imaging. We hypothesized that a superior drought tolerant NIL would be the one that performs better than Pusa 44 during drought stress while showing a less significant reduction in the projected shoot area (PSA), a slower increase in NIR intensity as well as a maximum reduction in water use (WU) and transpiration rate (TR). Agromorphologic data were also recorded manually at the time of harvest from all the test genotypes for comparative evaluation.

## Materials and Methods

### Plant Materials

A subset consisting of 35 promising NILs was identified from a population of 143 di-QTL introgression lines carrying two RSDS tolerant QTLs, *qDTY2.1* and *qDTY3.1*, in the background of Pusa 44. The subset was selected based on agronomic performance and grain quality parameters. Additionally, four genotypes were used as checks, which included, Pusa 44 – the recipient parent (RP), IR81896-B-B-142 – the donor parent (DP), and two RSDS tolerant checks *viz.*, IR81896-B-B-195 (RC1) and IR87728-59-B-B (RC2). Of these, Pusa 44 is a popular rice variety of north-western India suitable to the rice-wheat cropping system, with good yield, grain quality, and ideal agronomic characteristics. The donor, IR81896-B-B-142, is a line developed from the cross Apo/Swarna*2 carrying both the QTLs: *qDTY2.1* and *qDTY3.1* (Venuprasad et al., 2009). Among the checks, RC1 was developed from the same cross as that of the DP, while the second check, RC2, was derived from the cross, Aday sel/IR64. RC2 carries the QTLs *qDTY2.2* and *qDTY4.1* (Sandhu et al., 2018). The plant materials were grown with standard cultural practices for rice under transplanted conditions. Both the checks were proven to be field tolerant to RSDS from multiple experiments and locations (Singh et al., 2016; Dwivedi et al., 2021).

### Field Phenotyping for Drought Stress Response

**FIGURE 1:**
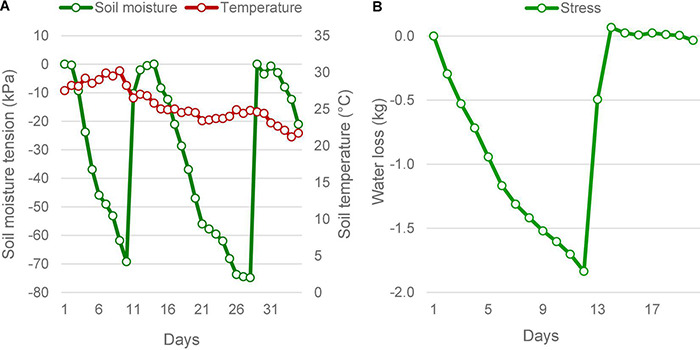
Soil moisture status under stress in the field and pot culture experiments. **(A)** In the field, soil moisture depletion was determined by the increasing soil moisture tension, measured using tensiometers. The soil moisture tension reached below -70 kPa twice during the reproductive stage, which forced lifesaving irrigations. Average ambient air temperature during the period did not vary much. It took an average of 9.5 days to reach the maximum stress from field capacity. **(B)** The pot culture under phenomics also was subjected to similar stress, the moisture depletion was measured as the falling weight of the pots. When the pots lost 1,840 g of water on the eleventh day, the plants experienced severe stress as that seen in the field, which was followed by lifesaving irrigation on the twelfth day of imposing stress.

The first field experiment was set up using 35 NILs grown in an augmented randomized block design (ARBD) with six blocks along with four checks. For each genotype, 120 plants were grown per treatment in four rows using a spacing of 20 × 15 cm. The NILs were subjected to two treatments, unstressed and stressed. In the unstressed treatment, a normal irrigation regime was maintained throughout the crop duration. Under stressed treatment, irrigation was discontinued after the initiation of the reproductive phase. However, lifesaving irrigation was provided when the soil water potential reached −70 KPa. Both the treatments received the same agronomic management, except for the imposition of drought stress (Figure 1). At the physiological maturity stage, five uniform looking plants were selected from each genotype per treatment for recording phenotypic data such as days to 50 percent flowering (DFF), plant height (PH), number of productive tillers (NPT), panicle length (PL), biomass (BM), spikelet fertility (SF), grain yield per plant (YP), hulling percentage (H%), milling percentage (M%), yield per hectare (PY), the weight of 1,000 grains (TW), and elongation ratio (ER). The experiment was carried out at the research farm of the Division of Genetics, ICAR-Indian Agricultural Research Institute (ICAR-IARI), New Delhi. In the next season, nine selected best NILs were field evaluated in a randomized complete block design (RCBD) along with the same four checks.

### Controlled Environmental Phenotyping

A pot culture experiment was conducted using 35 NILs along with their parents, Pusa 44 and IR 81896-B-B-142, in a controlled environment facility at the Nanaji Deshmukh Plant Phenomics Centre (NDPPC) at ICAR-IARI, New Delhi. NILs were initially sown in a raised bed nursery and transplanted after 30 days into the field. At the active tillering stage (∼ 30 days after transplanting), the plants were shifted to pots filled with 15 Kg of soil. Care was taken to keep the soil core intact without damaging the roots. After planting, the pots were flood irrigated with an equal volume of water (∼3 liters) and the weight on the pots was equated to 18.5 kg using soil. Pots were allowed for 12 h to get the soil fully saturated. The next day, all the pots were weighed again to confirm the uniformity in pot weight. The pot weight was measured using an automated weighing station. The potted plants were then shifted to the climate-controlled greenhouses at the NDPPC. A total of 156 pots were maintained, with two plants per treatment for each genotype. All the pots were tagged according to the cars they were loaded to. The plants were irrigated twice a day at 6 AM and 6 PM up to saturation pot weight and maintained well-watered with full saturation of 25% w/v soil moisture content (SMC). First, imaging of the plants was done 80 days after sowing. Thereafter, irrigation for one set of pots (stressed treatment) was restricted till the soil moisture dropped to 50% of saturation coinciding ∼ 12 % SMC. At this stage, all the plants showed severe drought symptoms and the SMC was estimated through the gravimetric method. Lifesaving irrigation was given at this stage to reach the saturation pot weight, raising the SMC to 25%. During the interval between the withdrawal of irrigation and the saturation, daily pot weight data was collected for measuring the progress of water depletion in the drought-stressed pots. From this data, the evapotranspiration (ET) was computed using the pot weight difference between the consecutive days. A set of pots without plants (mocks) were used as the internal control for measuring evaporation (E), using which whole plant transpiration per pot (T) was calculated and expressed in grams per pot. WU was also measured from the water consumption data and recorded in ml/g/day. The potted rice plants were maintained in a climate control led greenhouse, providing a photo regimen of 14 h light/10 h dark, a temperature regimen of 32/25*^o^*C day/night, and relative humidity of ∼ 60%.

Imaging of the genotypes was carried out for five-time snaps with five days’ intervals using RGB and NIR sensors. These stages, represented as I, II, III, IV, and V, indicated the number of five-day intervals. Stages I, II, and III denoted the phases under drought stress, after withholding the irrigation, while the stages IV and V denoted the recovery phase after providing the life-saving irrigation. The RGB images were captured using a Prosilica GT6400 (Allied Vision, Stadtroda, Germany) series visual camera with an RGB spectral range of 400-700 nm installed in Scanalyzer3D imaging platform (Lemna Tec GmbH, Aachen, Germany). Images were captured from one top view and three side views (SV0, SV120, and SV240) having a pixel matrix of 6576 × 4384. The raw images were acquired with the basic setup of the camera with an exposure set to 30,000, gain set to 20, gamma set to 125, the red-white balance of 110, and blue-white-red balance of 195, and provided with a constant fluorescent light source. The raw images were processed using the LemnaGrid^®^ software (LemnaTec GmbH, Aachen, Germany) using standard image processing tools. RGB images captured from all the three side views were used to estimate the PSA of the plant and expressed in cm^2^ (Neilson et al., 2015; Parent et al., 2015). The estimated PSA was used for normalizing whole plant transpiration to calculate the transpiration rate (TR) and expressed in g per cm^2^. To capture the leaf architectural changes specific to drought stress response, the stage-wise PSA was normalized with tiller number and expressed as PSA per tiller. The RGB data was also used to estimate a projected plant area (PPA), which was used for computing the growth rate. The rate of growth was calculated by dividing the PPA by the time interval in days between the two measurements. The hydration status of the plant was measured using NIR imaging. A Goldeye^®^ G032 NIR camera (AVT Allied Vision, Exton, Pennsylvania, United States) with a pixels’ resolution of 636 × 508 was used to acquire NIR images at 0°, 120°, and 240° from the side-view. To delineate the region of interest for the stress response, the NIR gray images were matched with corresponding RGB images. However, to compensate for the differences in the plant positions and in the image resolution between NIR and RGB images, a local matching method using 130 matching points was used. The matched images were cropped to a uniform size, and the plant area calculated from the RGB image was extracted to confirm the average water content. The average NIR pixel intensities were obtained at the water absorption wavelength of 1,450 nm, which was read within a range of zero to 255. Therefore, plants with a high-water content showed a low NIR intensity. The mean gray value was used for easy assessment of plant water status. At the time of maturity, the plants of individual pots were harvested and final phenotypic data was recorded, for traits namely PH, BM, TN, and YP.

### Calculation of Drought Indices

To assess the response for grain yield under drought, nine stress indices were calculated for both pot and field experiments. These indices were computed for each genotype using two parameters, yield under unstressed (Y_p_) and yield under stressed conditions (Y_s_). The average grain yield of all genotypes under unstress and stress conditions were represented by Ỹ_p_ and Ỹ_s_, respectively. Indices were used for initially ranking the NILs for each index based on the key drought tolerance indicator attached to it. Details of the indices and their key indicators for drought tolerance are given in Table 1.

**TABLE 1:** Drought tolerance indices used in the study.

Drought tolerance index	Notation	Formula	Unit	Range	Tolerance indicators	References
Relative stress tolerance	TOL	100(Y_p_–Y_s_)/Y_p_	%	0–100%	A low value indicates tolerance	Rosielle and Hamblin (1981)
Mean productivity	MP	0.5(Y_p_+Y_s_)	g	NIL	A high value indicates a better yield	Rosielle and Hamblin (1981)
Geometric mean productivity	GMP	(Y_p_*Y_s_)^0.5^	g	NIL	Similar to MP	Fernandez (1992)
Stress index	SI	1–(Ỹ_s_/Ỹ_p_)	−	0.0 – 1.0	A low value indicates mild stress or high mean tolerance	Fischer and Maurer (1978)
Stress susceptibility index	SSI	[(1–(Y_s_/Y_p_)]/SI	−	0.0 – 1.0	A low value indicates relatively high tolerance over the average stress	Fischer and Maurer (1978)
Stress tolerance index	STI	(Y_p_*Y_s_)/(Ỹ_p_)^2^	−	0.0 – 1.0	A high value indicates better tolerance	Fernandez (1992)
Drought yield index	YI	Y_s_/Ỹ_s_	−	NIL	A high value indicates better tolerance	Lin et al. (1986)
Yield stability index	YSI	Y_s_/Y_p_	−	0 – 1.0	A high value indicates better tolerance	Bouslama and Schapaugh (1984)
Relative tolerance	RT	(Y_p_–Y_s_)/Y_s_*100	%	0 – 100	A low value indicates tolerance	Oo et al. (2021b)

### Data Processing and Statistical Analyses

The data were analyzed for variability and trend over the experimental period. Additionally, Pearson’s correlations were worked out between drought indices and phenomics parameters. All the basic data operations and elemental statistical analyses were carried out using the Data Analysis Addin in Microsoft Excel^®^ v. 2019 (Microsoft Corporation, Redmond, United States). The agromorphologic data from the field and pot experiments were subjected to analysis of variance, carried out using STAR package 2.0.1 (IRRI, 2014). For the grain yield, statistical comparisons were done under stressed and unstressed treatments.

## Results

### Intensity of Drought

In both field and pot experiments, the intensity of drought reached critical levels producing significant drought responses in plants (Figure 1). In the field conditions, soil moisture level dropped twice to critical level falling to a maximum tension of −74.8 kPa, within a 35 day-window during the reproductive phase. The moisture depletion took 10 days to reach the critical level of −70 kPa from saturation during both episodes. During the 2016 trial, despite two peak drought incidences, there was only one lifesaving irrigation provided, due to a rain of 37.8 mm received on the tenth day. This rain did not affect the experiment, because by the time the soil moisture tension had reached −69.3 kPa. However, during the 2017 season, two lifesaving irrigations were needed, as there was no rain during the reproductive stage (Supplementary Table 1). Following the lifesaving irrigation, the moisture level was restored to saturation. The ambient average temperature during this period did not show much variation but had shown a dropping trend as the days advanced. The temperature was maximum on the 9*^th^* day with an average of 30.2°C. Under the pot experiment, soil moisture drop was induced only once, which showed a similar pattern of depletion as observed in the field. However, it took 12 days to reach the 50% depletion level. At this point, the average SMC ranged between 10.01 and 13.80 %, under gravimetric determination. There was no variation in the ambient temperature under pot culture.

### Variability of Near Isogenic Lines’ Response Under Imposed Drought

The ANOVA revealed significant variations for yield and related agronomic traits under both stress and unstress conditions in the field as well as pot-based evaluation (Table 2). The variation among the NILs was higher for most of the traits under stress conditions than those recorded under unstressed treatment. The ratio of stress and unstress variances indicated higher variation for NPT, H%, M%, ER, and SF. However, the variation among NILs was found to be higher in unstress conditions for a few traits like DFF, PH, and TW. The variance for BM and PY were relatively similar under both conditions in the field. Whereas, under the pot culture significant variation could be observed only for for NP and YP among the NILs and checks under stress. But, significant variation could be noticed only for YP under unstressed treatment in this experiment. The ratio of variances also revealed inconspicuous differences.

**TABLE 2:** The ANOVA for agronomic traits showing components of variance under stressed and unstressed treatments in the near-isogenic lines (NILs) in the field trial.

Trait	Variance under stress (Vs)			Variance under unstress (Vus)			Vs/Vus for NILs
	Checks	NILs	Residual	Checks	NILs	Residual	
DF*_f_*	144.0*	2.2*	54.4*	121.0*	13.3*	42.71*	0.17
PH*_f_*	1354.0*	9.0*	750.5*	1253.0*	20.0*	1261.9*	0.45
NP*_f_*	0.2*	101.1*	6.9*	2.6*	1.6*	1.1*	62.62
PL*_f_*	0.4*	1.0*	0.3*	0.04*	1.5*	7.6*	0.68
BM*_f_*	24.6*	83.0*	161.1*	161.8*	90.3*	39.4*	0.92
SF*_f_*	8.8*	64.5*	301.0*	8.6*	44.1*	385.4*	1.46
YP*_f_*	9.6*	10.5*	69.5*	3.2*	19.5*	46.2*	0.54
H%*_f_*	6.0*	223.8*	161.6*	1.7*	1.3*	1.2*	178.18
M%*_f_*	3.1*	163.3*	241.3*	3.2*	1.7*	0.1*	96.74
TW*_f_*	15.7*	1.8*	9.2*	0.3*	3.5*	5.9*	0.52
PY*_f_*	1,058,244*	1,158,970*	4,108,996*	777,060*	1,158,038*	29,022,097*	1.00
ER*_f_*	0.01*	0.01*	0.02*	0.00*	0.001*	0.01*	8.00
PH*_p_*	419.1*	18.6^ns^	1537.9*	619.5*	19.2^ns^	685.6*	0.97
W1*_p_*	79.3^ns^	26.4^ns^	7.7^ns^	93.9^ns^	50.0^ns^	590.6^ns^	0.53
W2*_p_*	73.4*	9.9^ns^	21.8^ns^	72.9^ns^	7.9^ns^	117.5^ns^	1.25
BM*_p_*	195.4^ns^	46.3^ns^	67.4^ns^	275.5^ns^	61.9^ns^	1234.9^ns^	0.75
NP*_p_*	6.8*	2.1*	0.3^ns^	0.3^ns^	1.1^ns^	11.3^ns^	1.79
YP*_p_*	9.5*	3.8*	20.0*	3.0*	3.6*	19.8	1.05

DF, days to 50% flowering; PH, plant height in cm; NP, number of panicle-bearing tillers; PL, length of panicle in cm; BM, biomass per plant in g; W1, the weight of plant part above the pot in g; W2, the weight of plant part between pot and soil level in g; SF, spikelet fertility in %; YP, grain yield per plant in g; H, hulling per cent; M, milling percentage; TW, weight of 1000 grains in g; PY, Plot yield in kg per ha; ER, elongation ratio; *significant at 5% level. The suffixes f and p indicate field and pot culture, respectively; ns, non-significant.

### Image-Based Phenotyping of Near Isogenic Lines Under Stressed and Unstressed Conditions

**FIGURE 2:**
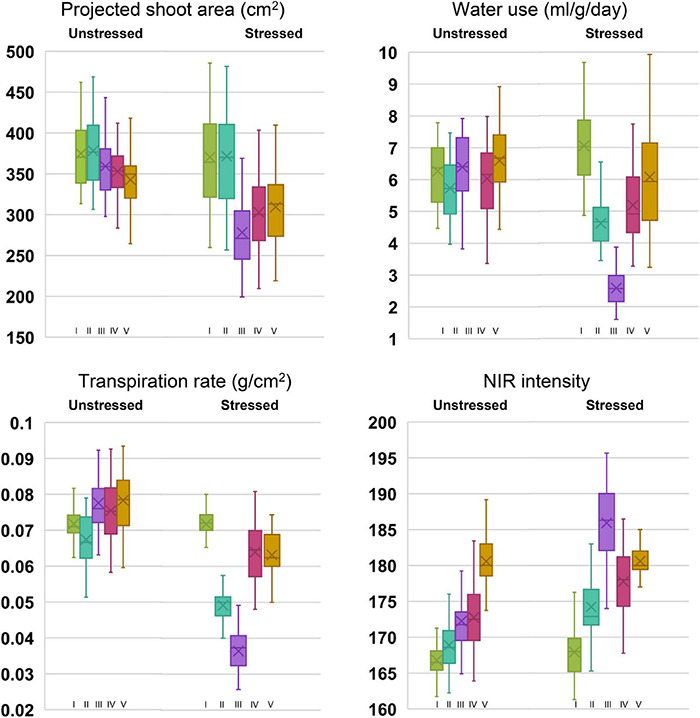
Box plots of phenomics parameters, projected shoot area (PSA), water use (WU), transpiration rate (TR), and near-infrared values (NIR) among the genotypes under stressed and unstressed treatments for five stages of drought exposure. Stages I to V corresponds to the initial phase without drought (0 days), 5 days after withholding irrigation in drought set, peak drought phase at 10 days after drought imposition when soil moisture content reached 12%, two days after irrigation, and 5 days into the recovery phase, respectively.

Under controlled phenotyping, four major traits (PSA, WU, TR, and NIR intensity) estimated from RGB and NIR images signified the effect of drought at various test stages. The box and whisker plots for traits, indicated relatively lesser variability for all the traits under unstressed conditions than under stress (Figure 2). Compared with the response under unstressed pots, an apparent drop was observed for stage-wise PSA, WU, and TR in the stressed pots. The drop occurred till stage III, beyond which the traits showed improvement, thereby producing a positive trend following irrigation. In the case of NIR intensity, under drought, the mean gray values increased with progressive stress, which declined at the recovery phase. Analyzing the mean performance of treatment and genotypes at different stages of the trial, it was noticed that the measured traits were statistically on par before the stress initialization (stage I). As the trial progressed, the trait means began to show significant statistical differences among the plants that were subjected to stress (Supplementary Table 2). For traits such as WU, TR, and NIR, apparent treatment effects could be detected beginning from stage II. The treatment difference for PSA was found remarkable beginning from stage III. Further, the differences in treatment effect could be seen even at the recovery stages after irrigation among the traits such as PSA, WU, and TR. The NIR values, however, showed a quick reversal during the recovery phase (stage V) by indicating non-significant differences among the treatment means. The genotypic variability was also found to vary with the stress treatment. We could observe significant variations among the parents and the NILs for PSA beginning from stage III. Whereas, for WU and TR, the genotypic differences became apparent only beyond stage IV. Interestingly, the differences in NIR values could be noticed only during stage IV. The average treatment values for PSA, WU, and TR were higher under irrigated treatment than the stressed one. Among the parental lines, under both stressed and unstressed conditions, higher PSA, WU, and NIR intensity were observed for the donor parent, IR81896-B-B-142, over the recurrent parent, Pusa 44. The NILs, however, showed significant improvement over Pusa 44 for these traits. In the case of NIR intensity, the mean values steadily increased from stage I (166.81) to stage V (180.7) under unstressed pots. When subjected to stress, the intensity initially increased from 167.56 (stage I) to 185.62 (stage III) and recovered back to 180.54 at stage V. The rate of transpiration decreased significantly from 0.072 to 0.036 and recovered up to 0.063 g/cm^2^ under stress conditions. The coefficient of variation (CV) between the stages for each genotypic class showed a conspicuous increase in the CV between stressed and unstressed treatments. Prominent CV difference was observed for traits such as WU, TR, and NIR.

Comparing the individual genotypic response, under drought stress, all the NILs were found to perform better than Pusa 44, while several of them outperformed the donor parent, IR 81896-B-B-142 (Supplementary Table 3). Although there was a reduction in parameter estimates of PSA, WU, and TR as the drought treatment progressed through stages I to III, NILs along with the donor parent generally showed a rapid recovery than Pusa 44. For PSA, the percentage reduction among the NILs from stage I to stage III was ranging between 9.1 and 33.7%. The WU and TR (Supplementary Tables 4, 5) were significantly reduced from stage I to stage III and ranged between 44 to 76.1% and 25.9 to 67.4%, respectively. Similarly, NIR intensity values were found to increase during drought stress and percent increase ranged between 3.5 and 14.6 % (Supplementary Table 6). Out of 35 NILs, four lines (P1823-12-42, P1823-12-82, P1823-12-118, and P1823-12-143) were found to be superior with a lesser reduction in PSA; lesser increase in NIR intensity with the highest reduction in WU and TR during progressive drought stress (Stage I to III). A few additional NILs (P1823-12-21, P1823-12-44, P1823-12-48, P1823-12-49, P1823-12-50, P1823-12-63, P1823-12-77, P1823-12-79, P1823-12-80, and P1823-12-81) were also found good as they showed a lesser reduction in PSA, TR, and WU along with a lesser increase in NIR intensity.

We could identify NILs with rapid recovery, another key indicator of drought tolerance. These lines showed a rapid gain in PSA, WU and TR while having a rapid reduction in NIR values. The post irrigation PSA increase at stage III among the NILs ranged between 0.4 and 23.7 %, as against the 20.6% of the donor parent, IR81896-B-B-142. PSA increment in Pusa 44 was 11.2%. Among the NILs, P1823-12-65, P1823-12-114, P1823-12-77, P1823-12-48, P1823-12-64, P1823-12-50, P1823-12-32, P1823-12-141, P1823-12-42, P1823-12-23, P1823-12-49, P1823-12-123, P1823-12-98, P1823-12-127, P1823-12-63, and P1823-12-104 were identified to perform better than Pusa 44. The values for WU and TR were also found to increase from stage I to stage III in all NILs between 31.8 to 71.2 % and 18.1 to 55.8 %, respectively. Where, higher WU and TR values were observed in the donor parent (63.8% and 50.8%, respectively) than in Pusa 44 (59.6% and 41.8 %). The NILs such as, P1823-12-23, P1823-12-32, P1823-12-42, P1823-12-48, P1823-12-64, P1823-12-65, P1823-12-84, P1823-12-104, P1823-12-118, P1823-12-124, and P1823-12-130 were found to have better WU as well as TR than Pusa 44. The reduction in NIR intensity values during the post-stress recovery period ranged between 0.2 and 6.7% among the genotypes. The highest reduction was observed in IR81896-B-B-142 (6.0%) than in Pusa 44 (4.2%). The NILs which indicated better recovery through a rapid decline in NIR intensity included P1823-12-32, P1823-12-48, P1823-12-65, P1823-12-80, P1823-12-96, P1823-12-104, P1823-12-124, P1823-12-132, and P1823-12-141, which was better than Pusa 44.

### Temporal Responses of Parents and Near Isogenic Lines

**FIGURE 3:**
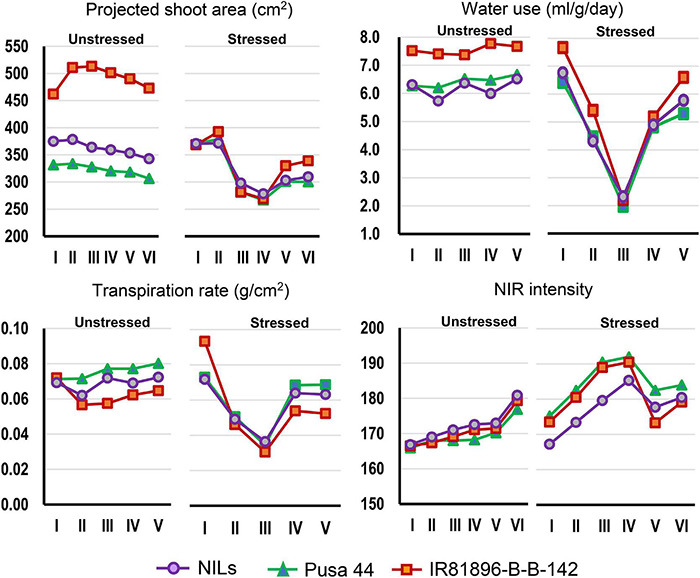
Graphical comparison of the temporal response of genotypes under stressed and unstressed treatments for the four phenomics parameters. The overall trend indicated differential response under stress which was largely absent under unstressed conditions.

Graphical comparison of the genotypic response pattern over the five stages of phenotyping, indicated significant contrast between the parents for traits such as PSA, WU, TR, and NIR under unstressed conditions (Figure 3). However, the response curves were largely flat indicating inconspicuous differences between the stages. In contrast, under drought, all the genotypes showed a common response pattern with a declining phase during progressive drought and a recovery phase after irrigation as described earlier. Interestingly, the NILs were found almost similar to Pusa 44 under unstressed conditions, while they exhibited a significant shift away from Pusa 44 under drought. Particularly for NIR intensity, NILs showed a lower value when compared with Pusa 44, indicating less water use to maintain higher leaf hydration status.

**FIGURE 4:**
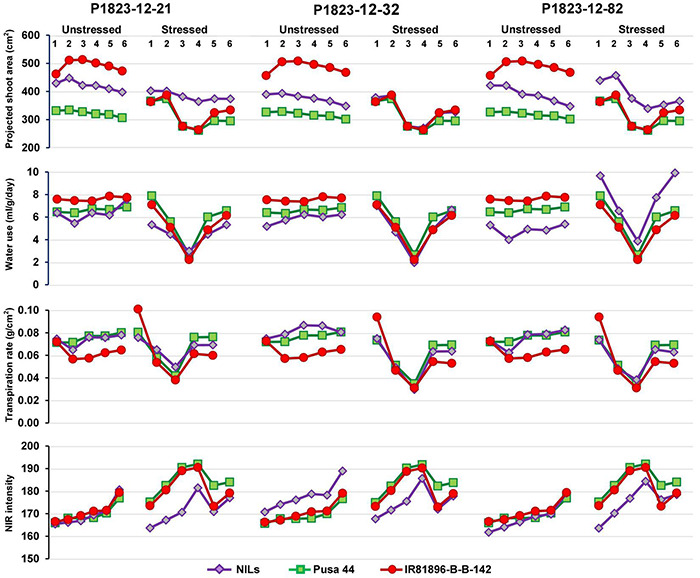
Graphical trends of three variable near-isogenic lines (NILs) (P1823-12-21, P1823-12-32, and P1823-12-82) under control and stress conditions for phenomics parameters.

Differential responses were recorded for some of the NILs in terms of their values at various stages. Out of the 35 NILs evaluated, 14 NILs were showing desirable trends for all the four parameters both under stress and non-stress conditions. While some of the lines showed a flatter curve during drought, indicating their endurance, while others showed a trend parallel to the donor parent. These criteria were used for selecting the best-performing ones. Some NILs outperformed both the parents under drought conditions and while remaining similar to Pusa 44 during unstressed conditions. Based on these patterns, three NILs, namely, P1823-12-21, P1823-12-32, and P1823-12-82 which showed tangible differences in the trend were selected for further comparison (Figure 4).

Under non-stress, all the three NILs showed a near similar trend for all the traits, with a clear advantage over Pusa 44. Under stress, P1823-12-21 showed a nearly flat trend for PSA along with low WU and NIR, and an intermediate TR between the parents. While under stress, this NIL showed a very similar pattern for PSA, WU, and TR, like that of both the parents. The NIR values of P1823-12-21 were lower than the parents. On the contrary, P1823-12-32 did not show differences in PSA values at all stages under stress from the parents. This NIL also showed no significant variation in WU and TR, except for a low NIR intensity in the initial stages of stress indicating better maintenance of systemic hydration status. The third NIL, P1823-12-82 showed significantly improved PSA and WU under stress with low NIR intensity and an intermediate TR that was apparent during the recovery phase. Accordingly, P1823-12-82 was considered to have the best drought response followed by P1823-12-32 and P1823-12-21. All the NILs showed trends similar to that of the three selected NILs for different parameters. The line graphs for all other NILs are presented in Supplementary Figure 1.

### Agronomic Performance of the Near Isogenic Lines Under Controlled Environment Screen

Following the controlled drought exposure under the phenomics platform, the agronomic traits of the genotypes were compared for the agronomic performance (Table 3). The results indicated significant differences among the stressed and unstressed treatments as well as within the genotypes. Considering the CV, stressed treatment showed a larger variation for NT and YP relative to unstressed treatment. However, PH and PB did not show explicit variation at the maturity stage. Further, the variation for TN was also not much remarkable as that of YP. Therefore, among the traits, grain yield was the trait largely affected by the transient drought treatment provided at the reproductive stage. Comparing the relative yield reduction (RDY) between the NILs and parents could bring out the effect of drought on the genotypes. The RDY values for the NILs ranged between 0.0 and 0.73 with an average of 0.34. The recurrent parent, Pusa 44 showed an RDY of 0.71, while IR 81896-B-B-142 had that of 0.49. This indicated that most of the NILs performed better than the parent, under stress. Seven NILs, P1823-12-23, P1823-12-48, P1823-12-63, P1823-12-64, P1823-12-65, P1823-12-114, and P1823-12-141 possessed higher RDY values than the tolerant parents IR81896-B-B-142. The rest of the 28 NILs were significantly better than both the parents under stress. One of the NILs, P1823-12-89 produced equal yield under both stress and unstressed conditions bringing the RDY to zero. Similarly, there were two other NILs, P1823-12-104 and P1823-12-122, which had RDY less than 10%. Among the 35 NILs tested, 34 of them have RDY values above that of Pusa 44, while 28 exceeded the donor parent. Also, the relative tolerance (RT) was compared for the 28 NILs that performed better than both parents, and the RT values were found to range from 0 to 87. RT of tolerant (donor) parent IR81896-B-B-142 was 100, while that of Pusa 44 was 256. Based on the pattern of drought response that emerged from the controlled phenotyping, the NILs’ performance was further compared to their actual field performance to establish any correspondence.

**TABLE 3:** The average performance of NILs and their relative tolerance under stress in the pot experiment under drought stress and unstressed conditions.

Genotypes	Unstressed				Stressed				RDY	RT
	PH	PB	NT	YP	PH	PB	NT	YP		
P1823-12-4	84.0^cd^	58.6^c^	13.0^b^	13.0^bc^	86.0^ab^	61.1^a^	12.0^b^	7.5^d^	0.42	73.3
P1823-12-21	76.0^k^	50.2^f–h^	10.0^e^	7.5^m^	74.0^ij^	47.0^e–h^	9.0^e^	6.0^ef^	0.20	25.0
P1823-12-23	82.0^ef^	53.4^de^	10.0^e^	10.0^hi^	74.0^ij^	47.0^e–h^	9.0^e^	4.0^ij^	0.60	150.0
P1823-12-32	82.0^ef^	47.8^h–l^	11.0^d^	8.0^lm^	79.0^de^	40.7^kl^	10.0^d^	6.5^e^	0.19	23.1
P1823-12-36	80.0^gh^	77.2^a^	14.0^a^	10.5^gh^	70.0^mn^	51.3^c^	13.0^a^	6.5^e^	0.38	61.5
P1823-12-42	83.0^de^	40.9^st^	11.0^d^	7.5^m^	71.0^lm^	37.9^mn^	10.0^d^	5.5^fg^	0.27	36.4
P1823-12-44	76.0^k^	42.7^p–s^	11.0^d^	8.0^lm^	65.0^q^	37.3^mn^	10.0^d^	6.0^ef^	0.25	33.3
P1823-12-48	84.0^cd^	48.7^g–j^	12.0^c^	10.5^gh^	74.0^ij^	32.6^q^	10.0^d^	4.0^ij^	0.62	163.0
P1823-12-49	85.0^bc^	49.7^f–j^	12.0^c^	14.0^a^	75.0^hi^	48.7^def^	13.0^a^	8.5^bc^	0.39	64.7
P1823-12-50	79.0^hi^	42.7^p–s^	11.0^d^	13.5^ab^	77.0^fg^	32.1^q^	9.0^e^	9.0^ab^	0.33	50.0
P1823-12-63	84.0^cd^	43.1^o–s^	10.0^e^	11.5^ef^	71.0^lm^	45.7^ghi^	11.0^c^	4.5^hi^	0.61	156.0
P1823-12-64	85.0^bc^	47.2^i–m^	13.0^b^	9.0^jk^	71.0^lm^	40.3^l^	12.0^b^	3.0^k^	0.67	200.0
P1823-12-65	85.0^bc^	45.3^l–p^	13.0^b^	11.0^fg^	76.0^gh^	39.4^lm^	12.0^b^	3.0^k^	0.73	267.0
P1823-12-77	82.0^ef^	49.8^f–i^	12.0^c^	8.5^kl^	81.0^c^	40.9^kl^	12.0^b^	6.0^ef^	0.29	41.7
P1823-12-79	85.0^bc^	45.5^l–o^	11.0^d^	9.0^jk^	80.0^cd^	44.8^hij^	13.0^a^	8.0^cd^	0.11	12.5
P1823-12-80	75.0^kl^	32.1^u^	10.0^e^	7.5^m^	77.0^fg^	44.1^ij^	12.0^b^	4.0^ij^	0.47	87.5
P1823-12-81	86.0^b^	53.9^de^	13.0^b^	9.5^ij^	85.0^b^	54.5^b^	13.0^a^	7.5^d^	0.21	26.7
P1823-12-82	81.0^fg^	55.8^d^	11.0^d^	11.5^ef^	79.0^de^	33.6^pq^	10.0^d^	8.5^bc^	0.26	35.3
P1823-12-84	83.0^de^	54.3^de^	12.0^c^	9.0^jk^	73.0^jk^	45.5^ghi^	10.0^d^	5.0^gh^	0.44	80.0
P1823-12-89	71.0^m^	48.6^g–j^	12.0^c^	9.5^ij^	69.0^no^	49.3^cde^	13.0^a^	9.5^a^	0.00	0.0
P1823-12-96	84.0^cd^	45.2^m–p^	12.0^c^	11.0^fg^	73.0^jk^	50.5^cd^	13.0^a^	8.0^cd^	0.27	37.5
P1823-12-98	78.0^ij^	38.7^t^	12.0^c^	12.5^cd^	78.0^ef^	38.6^lm^	12.0^b^	8.5^bc^	0.32	47.1
P1823-12-104	70.0^mn^	42.6^q–s^	12.0^c^	10.0^hi^	76.0^gh^	46.0^ghi^	13.0^a^	9.0^ab^	0.10	11.1
P1823-12-114	81.0^fg^	44.5^n–r^	12.0^c^	12.0^de^	80.0^cd^	37.7^mn^	11.0^c^	5.5^fg^	0.54	118.0
P1823-12-118	86.0^b^	45.0^m–q^	10.0^e^	8.5^kl^	73.0^jk^	54.9^b^	10.0^d^	5.5^fg^	0.35	54.5
P1823-12-120	78.0^ij^	64.9^b^	14.0^a^	12.0^de^	71.0^lm^	46.6^fgh^	12.0^b^	9.5^a^	0.21	26.3
P1823-12-122	80.0^gh^	44.1^n–r^	11.0^d^	9.0^jk^	80.0^cd^	42.6^jk^	9.0^e^	8.5^bc^	0.06	5.9
P1823-12-123	81.0^fg^	42.3^rs^	11.0^d^	11.5^ef^	77.0^fg^	36.0^no^	9.0^e^	9.0^ab^	0.22	27.8
P1823-12-124	76.0^k^	52.1^ef^	12.0^c^	12.5^cd^	71.0^lm^	39.2^lm^	10.0^d^	8.0^cd^	0.36	56.3
P1823-12-127	78.0^ij^	42.6^q–s^	12.0^c^	8.0^lm^	75.0^hi^	44.7^hij^	10.0^d^	4.5^hi^	0.44	77.8
P1823-12-130	75.0^kl^	48.2^g–k^	12.0^c^	8.5^kl^	68.0^op^	40.3^l^	10.0^d^	6.5^e^	0.24	30.8
P1823-12-132	86.0^b^	42.9^o–s^	11.0^d^	8.5^kl^	80.0^cd^	48.8^def^	9.0^e^	6.5^e^	0.24	30.8
P1823-12-134	80.0^gh^	48.8^g–j^	11.0^d^	7.5^m^	74.0^ij^	42.8^jk^	11.0^c^	6.0^ef^	0.20	25.0
P1823-12-141	69.0^n^	50.7^fg^	12.0^c^	8.5^kl^	72.0^kl^	34.0^opq^	12.0^b^	3.6^jk^	0.58	136.0
P1823-12-143	74.0^l^	45.1^m–q^	13.0^b^	9.0^jk^	81.0^c^	34.9^op^	12.0^b^	7.5^d^	0.17	20.0
Pusa 44	83.0^de^	45.7^k–n^	13.0^b^	8.0^lm^	78.0^ef^	35.8^nop^	11.0^c^	2.3^l^	0.71	256.0
IR81896-B-B-142	97.0^a^	47.1^j–m^	11.0^d^	8.5^kl^	87.0^a^	47.2^efg^	12.0^b^	4.3^hi^	0.49	100.0
SED	0.9	1.3	0.17	0.3	0.8	1.1	0.2	0.3	0.03	11.0
CD (0.05)	1.8	2.6	0.35	0.6	1.7	2.3	0.5	0.7	0.06	22.3
CV%	6.7	15.9	9.2	19.0	6.6	15.7	12.8	32.1		

*PH, plant height in cm; PB, plant biomass in g; NT, number of tilers; YPP, yield per plant in g; means suffixed with same letters are statistically not different by Tukey’s honestly significant different test.*

### Drought Indices

The pattern of drought tolerance indices divulged a better picture of the comparative evaluation of the drought tolerant NILs. Since these indices had different tolerance indicators emphasizing various drought responses, we have employed all of them together for evaluation. Seven drought indices calculated using yield per plant under control and stress conditions suggested that almost all the NILs performed better than recipient parent Pusa 44 in the field (Table 4). No indices were calculated for the pot experiment, as the drought imposed was not enough to dissect the tolerance response for grain yield, since only one episode of the drought was induced in this evaluation. The lower values of stress susceptibility index (SSI) and higher values of stress tolerance index (STI) indicated the better performance of the line under stress. All the NILs except five were showing lower values of SSI while 32 NILs had higher values for STI in comparison to Pusa 44 having an SSI was 1.56 and STI was 0.39. Corresponding SSI and STI values for IR81896-B-B-142 were 0.98 and 0.56, respectively.

**TABLE 4:** Drought indices and yield performance of Pusa 44 *qDTY* NILs in field experiment.

Entry	Yp	Ys	TOL	MP	GMP	SSI	STI	YI	YSI
Pusa44	22.2^wx^	10.9^s^	11.4^d^	16.6^r^	15.6^r^	1.6^ab^	0.4^pq^	0.7^t^	0.5^tu^
IR81896-B-B-142	22.4^v–x^	15.2^mno^	7.2^hij^	18.8^no^	18.4^o–q^	1.0^fgh^	0.6^klm^	0.9^mno^	0.7^k–n^
P1823-12-4	25.2^nop^	21.1^ab^	4.0^nop^	23.2^fgh^	23.1^f–h^	0.5^o^	0.9^d^	1.3^ab^	0.8^b^
P1823-12-21	19.5^z^	15.7^lmn^	3.7^op^	17.6^pqr^	17.5^p–r^	0.6^no^	0.5^mno^	1.0^lm^	0.8^bc^
P1823-12-23	29.0^e–i^	21.7^a^	7.3^hi^	25.3^bc^	25.1^bc^	0.8^kl^	1.0^b^	1.3^a^	0.8^e–h^
P1823-12-32	29.6^c–f^	14.0^opq^	15.6^ab^	21.8^ij^	20.4^ij^	1.6^a^	0.7^gh^	0.9^opq^	0.5^u^
P1823-12-36	28.7^e–j^	18.6^d–g^	10.0^def^	23.6^ef^	23.1^ef^	1.1^fg^	0.9^d^	1.1^e–h^	0.7^mno^
P1823-12-42	29.6^cde^	19.9^bcd^	9.7^f^	24.8^cd^	24.3^cd^	1.0^fgh^	1.0^b^	1.2^bcd^	0.7^lmn^
P1823-12-44	25.8^mn^	16.4^j–m^	9.4^f^	21.1^jk^	20.6^g–i^	1.1^ef^	0.7^g^	1.0^kl^	0.6^nop^
P1823-12-48	24.0^o–u^	16.6^i–m^	7.5^gh^	20.3^klm^	19.9^k–m^	1.0^hi^	0.7^ghij^	1.0^jkl^	0.7^j–m^
P1823-12-49	27.6^i–l^	21.2^ab^	6.4^h–k^	24.4^cde^	24.2^c–e^	0.7^k–n^	1.0^bc^	1.3^a^	0.8^c–g^
P1823-12-50	22.9^s–w^	17.2^h–k^	5.7^j–m^	20.1^k–n^	19.8^k–m^	0.8^kl^	0.7^gij^	1.0^ij^	0.8^e–h^
P1823-12-63	20.8^yz^	15.5^mn^	5.3^k–n^	18.2^opq^	18.0^p–r^	0.8^jkl^	0.5^l–n^	0.9^lmn^	0.7^f–i^
P1823-12-64	30.7^cd^	14.0^opq^	16.7^a^	22.3^ghi^	20.7^g–i^	1.7^a^	0.7^g^	0.8^o–r^	0.5^u^
P1823-12-65	29.6^c–g^	20.8^abc^	8.8^fg^	25.2^bc^	24.8^bc^	0.9^hij^	1.0^b^	1.3^abc^	0.7^i–l^
P1823-12-77	25.3^no^	11.5^rs^	13.8^c^	18.4^nop^	17.1^p–r^	1.7^a^	0.5^no^	0.7^t^	0.5^u^
P1823-12-79	33.6^a^	18.7^d–g^	15.0^bc^	26.2^ab^	25.1^ab^	1.4^cd^	1.0^b^	1.1^d–h^	0.6^rs^
P1823-12-80	24.3^o–s^	14.5^nop^	9.8^ef^	19.4^l–n^	18.7^no^	1.2^de^	0.6^jkl^	0.9^nop^	0.6^pqr^
P1823-12-81	29.5^c–h^	18.3^e–h^	11.2^de^	23.9^def^	23.3^d–f^	1.2^e^	0.9^cd^	1.1^f–i^	0.6^opq^
P1823-12-82	24.5^n–r^	19.3^def^	5.2^k–n^	21.9^ij^	21.8^ij^	0.7^lmn^	0.8^ef^	1.2^def^	0.8^cde^
P1823-12-84	16.5^z^	11.6^rs^	4.9^l–o^	14.01^s^	13.8^s^	0.9^hij^	0.3^r^	0.7^s^	0.7^i–l^
P1823-12-89	27.8^ijk^	13.2^pq^	14.6^bc^	20.5^kl^	19.1^k–m^	1.6^a^	0.6^ijk^	0.8^qrs^	0.5^u^
P1823-12-96	21.4^xy^	17.1^h–l^	4.3^no^	19.2^m–o^	19.1^k–m^	0.6^mno^	0.6^ijk^	1.0^ijk^	0.8^bcd^
P1823-12-98	24.2^o–t^	19.6^cde^	4.6^mno^	21.9^ij^	21.8^ij^	0.6^no^	0.8^ef^	1.2^de^	0.8^bc^
P1823-12-104	17.5^z^	12.7^qr^	4.8^l–o^	15.1^s^	14.9^r^	0.8^ijk^	0.4^qr^	0.8^rs^	0.7^h–k^
P1823-12-114	20.0^yz^	18.5^d–h^	1.5^q^	19.2^m–o^	19.2^k–m^	0.2^p^	0.6^h–k^	1.1^e–h^	0.9^a^
P1823-12-118	31.0^bc^	16.1^klm^	14.9^bc^	23.5^ef^	22.3^ef^	1.5^bc^	0.8^de^	1.0^kl^	0.5^st^
P1823-12-120	32.2^ab^	21.5^a^	10.7^de^	26.83^a^	26.3^a^	1.0^fgh^	1.1^a^	1.3^a^	0.7^lmn^
P1823-12-122	22.6^u–x^	17.7^g–j^	4.9^l–o^	20.1^k–m^	20.0^jk^	0.7^lmn^	0.7^ghi^	1.1^hij^	0.8^c–f^
P1823-12-123	23.8^p–v^	14^opq^	9.8^ef^	18.9^no^	18.3^o–q^	1.3^d^	0.6^klm^	0.8^o–r^	0.6^qr^
P1823-12-124	25.1^n–q^	19.1^d–g^	6.0^i–l^	22.1^h–j^	21.9^h–j^	0.7^klm^	0.8^ef^	1.2^d–g^	0.8^d–h^
P1823-12-127	20.0^yz^	17.9^f–i^	2.1^q^	19.0^no^	18.9^no^	0.3^p^	0.6^ijkl^	1.1^ghi^	0.9^ab^
P1823-12-130	22.2^wx^	19.8^bcd^	2.4^pq^	21.0^jk^	21.0^jk^	0.3^p^	0.7^fg^	1.2^cde^	0.9^ab^
P1823-12-132	26.8^klm^	19.6^cde^	7.2^hi^	23.2^fg^	23.0^fg^	0.8^jk^	0.9^d^	1.2^de^	0.7^g–j^
P1823-12-134	20.4^yz^	14.0^opq^	6.4^hijk^	17.2^qr^	16.9^qr^	1.0^ghi^	0.5^nop^	0.9^opq^	0.7^j–m^
P1823-12-141	16.8^z^	8.0^t^	8.8^fg^	12.4^t^	11.6^s^	1.6^ab^	0.2^s^	0.5^u^	0.5^tu^
P1823-12-143	20.5^yz^	13.4^pq^	7.2^hij^	17.0^r^	16.6^qr^	1.1^fg^	0.5^op^	0.9^p–s^	0.7^m–o^
SED	0.73	0.56	0.66	0.56	0.56	0.07	0.04	0.03	0.02
CD(0.05)	1.48	1.13	1.34	1.13	1.13	0.13	0.07	0.07	0.04

*Y_us_, yield per plant (g) under unstress; Y_s_, yield under stress; TOL, relative stress tolerance; MP, mean productivity; GMP, geometric mean productivity; SSI, stress susceptibility index; SI, stress index; STI, stress tolerance index; YI, yield index; means suffixed with same letters are statistically not different by Tukey’s honestly significant different test.*

A similar pattern was seen for all other indices too, with NILs having higher mean productivity (MP) and yield index (YI) than Pusa 44. Besides, there were 27 NILs with higher MP and 24 with higher YI than the donor parent. Among the NILs, SSI ranged between 0.22 and 1.67, while STI had a range of 0.22 to 1.13. The corresponding averages were 0.975 and 0.686, respectively. Similarly, the average MP of NILs was 20.65g with a range of 12.39g to 26.83g. The MP of the parents was 16.55g for Pusa 44 and 18.77g for IR81896-B-B-142. Relative stress tolerance (TOL) indicates the drought impact on yield, irrespective of its magnitude. In this study, the TOL for Pusa 44 was 11.35 while that of the tolerant (donor) parent was 7.18. Lower values of TOL indicates less reduction of yield under stress as compared to yield under normal unstressed condition. Among the NILs, 29 lines had lower TOL values than Pusa 44, while 16 among these outperformed IR81896-B-B-142 with lower TOL values. The lowest TOL of 1.45 was recorded for P1823-12-114, followed by P1823-12-127 with 2.10 and P1823-12-130 with 2.44. Based on agronomic performance, grain quality, and drought indices, the number of selected NILs was narrowed down and the final best selections came out to be 14 NILs.

### Correlation Analyses

Correlation analyses were carried out to explore the correspondence between yield, drought indices, and phenomics parameters. When all the 35 NILs were used for computing the relations between grain yield and phenomics parameters, we could strike only a few significant correlations. This could be due to difference among the NILs for phenomics traits. However, when NILs were shortlisted based on the distinct drought tolerance responses, significant correlations emerged from the selected NILs. Initially 14 NILs were shortlisted based on the phenomics data which was subsequently reduced to nine based on both and field and phenomics data. Significant correlations could be noticed between grain yields from field and pot experiments (Table 5).

**TABLE 5:** Correlations between grain yield, phenomics parameters, and major tolerance indices among the selected NILs.

Stages	Yield parameters		35 NILs				14 NILs					9 Selected NILs			

			PSA	WU	NIR	TR	PSA	WU	NIR	TR	TOL	PSA	WU	NIR	TR
I	PY*_f_*	**PY*_p_***	−0.06	0.02	−0.16	−0.18	0.13	0.17	−0.15	−0.10	0.40*	0.82*	0.63*	−0.52	0.16
II		0.40*	−0.08	−0.06	−0.22	−0.23	0.32	0.18	−0.22	−0.04		0.89*	0.50	−0.52	−0.65*
III			0.10	0.17	−0.22	0.00	0.55*	0.57*	−0.22	−0.42		0.79*	0.41	−0.35	−0.26
IV			0.05	0.17	−0.17	−0.20	0.48*	0.40*	−0.04	0.02		0.80*	0.47	−0.36	−0.48
V			−0.07	0.10	0.00	−0.13	0.46*	0.46*	−0.25	−0.22		0.77*	0.50	−0.32	−0.50
I	YP*_f_*	**YP*_p_***	0.06	−0.07	−0.09	−0.13	0.54*	−0.10	−0.05	−0.26	−0.51*	0.57	0.39	−0.46	0.21
II		0.52*	0.06	−0.20	−0.08	−0.17	0.20	−0.08	−0.34	−0.06		0.67*	0.36	−0.38	−0.70*
III			0.27	0.08	−0.06	0.03	0.56*	0.37	−0.44	−0.32		0.55	0.43	−0.18	−0.08
IV			0.21	0.00	0.03	−0.03	0.44	0.16	−0.37	−0.22		0.54	0.45	−0.28	−0.39
V			0.12	−0.03	0.02	−0.25	0.39	0.39	−0.28	−0.39		0.60*	0.50	−0.37	−0.51
I	YP*_p_*		0.06	−0.07	−0.09	−0.13	0.55*	0.49*	−0.32	−0.11	−	0.43	0.20	−0.32	0.24
II			0.06	−0.20	−0.08	−0.17	0.56*	0.18	−0.17	−0.40		0.44	−0.11	−0.39	−0.65*
III			0.27	0.08	−0.06	0.03	0.71*	0.38	−0.07	−0.03		0.67*	0.17	−0.32	0.11
IV			0.21	0.00	0.03	−0.03	0.67*	0.38	0.04	−0.10		0.66*	0.08	−0.19	0.33
V			0.12	−0.03	0.02	−0.25	0.68*	0.46*	0.11	−0.04		0.61*	0.27	−0.41	−0.29
I	TOL		0.04	0.13	0.11	0.04	−0.46*	−0.09	0.28	0.03	−	−0.32	−0.09	0.29	0.01
II			0.11	0.44*	0.21	0.07	−0.45	0.15	0.28	0.18		−0.31	0.17	0.50	0.23
III			−0.14	0.21	0.23	0.12	−0.62*	−0.06	0.24	0.13		−0.53	−0.04	0.53	0.11
IV			−0.11	0.39*	0.14	0.22	−0.60*	0.07	0.14	0.42		−0.48	0.14	0.41	0.33
V			0.08	0.30*	−0.02	0.12	−0.54*	−0.11	0.09	0.32		−0.35	−0.04	0.39	0.21
I	SSI		0.08	−0.11	−0.05	−0.22	−0.32	−0.11	0.10	−0.32	0.90*	−0.33	−0.11	0.09	−0.35
II			0.15	0.35*	−0.02	0.09	−0.33	0.07	0.07	0.22		−0.32	0.16	0.17	0.29
III			−0.03	0.07	0.00	0.16	−0.49*	−0.02	0.03	0.21		−0.40	0.27	0.17	0.44
IV			−0.03	0.07	−0.05	0.21	−0.48*	0.08	−0.03	0.49*		−0.38	0.40	0.08	0.65*
V			0.13	0.02	−0.06	0.13	−0.47*	−0.08	0.16	0.42		−0.30	0.23	0.32	0.41

YP, yield per plant in g; PY, plot yield in kg/ha; TOL, relative stress tolerance from the field; SSI, stress susceptibility index; STI; PSA, projected shoot area; WU, water use; NIR, near-infrared intensity; TR, transpiration rate; stages I-V are the imaging stage points from the phenomics platform. Stage I, 1 DAI (days after irrigation); stage II, 5DAI; stage III, 9DAI with peak drought stress, irrigated on the next day; stage IV, 2DAR (days after recovery); stage V, 5DAR; * significant at 5% level. The suffixes f and p indicate field and pot culture, respectively.

Correlations between the TOL and grain yield and TOL and SSI values showed the expected pattern. While grain yield and STI indicated a strong negative correlation with TOL, a positive association was found between SSI and TOL. The stage-wise correlations worked out between the field-based yield and phenomics parameters also showed significance. Positive correlations for PSA could be observed with yield in both pot and field experiments, particularly at stages after stress. This was also reflected in the significant negative correlations of PSA with tolerance indices such as TOL and SSI. Although not apparent, positive significant correlations with grain yield were also exhibited by WU at various stages. The correlation for NIR and TR was negative for grain yield but was not pronounced as that of PSA and WU. Similarly, strong positive correlations could be observed between TR and two drought indices, TOL and SSI. Since TOL and SSI are correlated positively and strongly (.90), both provided equal opportunity in assessing the drought-tolerant status of the NILs. Correlations of agronomic parameters under field evaluation showed no correspondence due to the different performance of NILs under both unstressed and stressed treatments (Supplementary Table 7).

## Discussion

Drought is a complex stress to breed against, since its nefarious effects are inconsistent across genotypes and environments. Drought occurs when plants suffer from inadequate uptake of water to meet the water loss through transpiration. Although there can be several factors that incite drought, the most common ones in the agricultural systems are scarcity of irrigation water and irregularity in precipitation coupled with high temperature. Unlike other stresses, drought’s impact on the plant community is wide and ruinous. When drought strikes, every plant in the community perceives drought in varying magnitude, effects of which can be seen commonly in agricultural fields as dynamic phenotypic expressions. Therefore, phenotyping for drought response is a cumbersome task that requires a large area and population, with timebound activities and destructive sampling, resulting in compromised precision. Besides, various intervening factors such as unpredicted rains and poor infrastructure, contribute more to unexplained variance. To address this problem, controlled environment facilities such as rainout shelters are often advocated in drought-related studies, wherein the interference of unpredicted rains can be avoided. However, rainout shelters are huge and require a lot of manual interventions to operate, still do not offer the expected precision in phenotyping, and involve destructive sampling. Although expensive, phenomics platforms are highly improvised facilities equipped with precision instruments to image and measure plants as they grow in a controlled environment glasshouse. Unlike field-based screening systems where plants grow in a static position, in the phenomics platform individual plants are dynamically positioned in specialized pots which can be moved and randomized to provide uniform growing conditions. Besides, the plants travel and are continuously phenotyped, thereby providing unparalleled precision, high throughput, and non-invasive sampling.

Adapted to a semi-aquatic environment, the rice crop consumes about 2,500 liters of water to produce one kilogram of rough rice (Bouman, 2009). Reducing the water supply to rice crops from its normal levels has a penalty attached because moisture stress can cause yield loss up to 70% under severe deprivation (Kumaraswamy and Shetty, 2016). However, genotypic differences do exist in the level of yield loss, which can be translated into the development of drought-tolerant rice varieties. A tolerant genotype can endure drought by yielding grains, where a sensitive cultivar normally fails. Recently, there are several QTLs identified in rice related to grain yield under drought stress, named with the prefix qDTY (Vinod et al., 2019). Currently, several of these QTLs are being integrated into mainstream cultivars for augmenting them with drought tolerance. In this study, we have used a set of NILs developed from Pusa 44, a mega-scale cultivar of northern India, harboring a combination of two qDTYs, *qDTY2.1*, and *qDTY3.1* (Dwivedi et al., 2021; Oo et al., 2021a).

There are already reports of the usage of image-based parameters for assessment of plant health under different stress conditions like drought, heat, salinity, etc. in various crops (Jones et al., 2002; Roy et al., 2011; Yonemaru and Morita, 2012; Hairmansis et al., 2014; Campbell et al., 2015; Humplík et al., 2015). However, the use of these parameters for assessing drought tolerance has been seldom experimented in rice. In this study, we have used 35 improved NILs of Pusa 44 to assess the feasibility of deploying a phenomics platform for drought tolerance assessment. NILs being genetically near-uniform, provide a greater opportunity to examine subtle response variations conditioned by the integrated QTLs/genes in comparison with the recurrent parent. Therefore, we have attempted to compare the image-based crop response parameters from the phenomics with field performance, to identify best performing NILs. This has provided us the opportunity to assess the critical relationship between the phenomics parameters and grain yield under drought.

Comparing the parental response to induced drought, we found that the donor line, IR81896-B-B-142 had higher values of PSA and WU over Pusa 44. IR81896-B-B-142 possessed long and broad leaves than Pusa 44 with a tall plant stature. Pusa 44 had higher NIR intensity values and TR indicating its inclination to lose internal water due to the elevated TR. Primarily, these observations indicated the tendency of Pusa 44 to fail under drought. As the drought progressed, there was a decline observed among all the traits, except for NIR intensity which showed a reverse trend. This was because NIR increased with reducing internal water, while PSA, WU, and TR showed reduction as the drought progressed. However, the pattern of PSA decline was found to be different in some of the NILs, which showed little variation in the PSA over the drought period. Coincidently, those NILs that showed a lesser decline in PSA also showed better grain yield. It should be inferred that plants that showed lesser shrinkage have a better mechanism to conserve internal water status, thereby being able to produce better yield (Pandey and Shukla, 2015). This made us rely upon PSA as a selection parameter, because it showed a consistent association with grain yield among the NILs that produced higher yield under drought in both field and pot culture experiments. PSA is a measure of biomass obtained from RGB images taken from three dimensions, top and two side views (Honsdorf et al., 2014). Under the drought treatment, the trend of PSA decline showed a sharp decrease until stage III which corresponded to the onset of drought. This is corroborated from the PSA pattern under unstressed conditions which remained almost the same between the imaging stages with only statistically insignificant differences. Reduction in PSA indicated that the plant biomass was shrinking due to the loss of water. However, this decline reflected only a transient architectural change rather than a physical biomass loss. Visual imaging captures drought-induced leaf rolling and wilting in the plants and predicts lowering PSA. This is the reason why the PSA started to revert during stages IV and V following the irrigation. Therefore, in this study, PSA decline recorded a temporal response to drought, indicating that image-based phenotyping was capable of capturing even minute variations in plant architecture. If a prolonged drought is provided, the PSA can accurately reflect actual biomass loss. A similar response for PSA has been reported earlier corresponding to progressive drought stress (Mir et al., 2019; Kim et al., 2020). Leaf rolling under water stress is an adaptive mechanism by plants to minimize water loss from leaves that helps in survival. During this process, stomatal closure occurs to reduce transpiration loss of water (Kim et al., 2020). This helps the plants to use internal water judiciously and to maintain higher relative water content and to decrease leaf drying. Maintenance of water homeostasis in plants requires lowering water loss while maintaining a steady uptake from the soil, although at a reduced pace.

Other parameters such as NIR intensity, WU, and TR could also be used for further evaluation of lines. There are earlier reports of the use of these parameters to assess drought tolerance in crops including rice (Gupta et al., 2012; Deshmukh et al., 2014; Kumar et al., 2015; Malinowska et al., 2017; Kim et al., 2020). Plant responses in favor of tolerance could be perceived from reduced TR and NIR values. While TR translates directly to the water loss from the leaves, NIR indicated the hydration status of the plants. Hydrated plants generally show lower gray values for the NIR intensity, which increases along with drying up as drought progresses. Near-IR spectrometry is a recognized tool for determining leaf water content in plants (Zhang et al., 2012; Jin et al., 2017). Water use which indicates the internal water replenishment status also showed a positive trend with grain yield among the high yielding NILs. The TR also showed a decreasing trend as the drought progressed, concordantly to the internal water depletion. With an increase in stress, mean values for PSA began to decrease at a lesser rate than changes in WU and TR, indicating that changes in PSA occur after the water loss. Therefore, a deep decline in any of these traits indicated a sensitive reaction, as the tolerant genotypes are expected to have a slower change. Further, tolerant genotypes showed quicker recovery than the sensitive ones, reverting to the original values following irrigation. In this study, we have seen that NILs responded rapidly for re-watering and entered into the recovery phase than the recurrent parent. However, we could observe an elevation in the NIR values as the recovery progressed perhaps due to the plants entering their maturity.

Although all the NILs possessed the same combinations of QTLs, *qDTY2.1* and *qDTY3.1*, there were variations among them for the degree of drought tolerance. However, these variations remained latent under field evaluation, since the final yield was the major trait used to evaluate the tolerance. Whereas in the phenomics evaluation, instead of the outcome, dynamic observation of the crop response was made under drought, which could unfurl subtle variations in the plant response. This was the reason for the absence of a meaningful correlation between phenomics variables and grain yield when the data from all the 35 NILs were used for computations. However, when the NILs were shortlisted based on the desired response pattern based on phenomics variables, the correlations became apparent and robust. Phenotypic deviations among the NILs introgressed with the same QTLs are not an uncommon feature in maker-assisted introgression breeding because of the transmission of untargeted donor loci. Although such segments may not produce perceptible phenotypic deviation from the recurrent parent, they can indulge in interactions with the background genome resulting in varying expression levels of the introgressed QTLs. Background interactions from the small introgressed segments have been reported to influence target gene/QTL expression under varying environments (Li et al., 2020).

We could establish that the NILs used in this study showed improved adaptation to drought than the recipient parent, Pusa 44, but with varying degrees of advantage. The phenomics parameters could, however, dissect these variations into a specific pattern with PSA, WU, TR, and NIR striking a significant correlation with grain yield. Among the varying patterns, the most desirable combination was a flatter curve for the PSA as seen in P1823-12-21, higher WU as found in P1823-12-82 coupled with low TR and NIR values. This combination can be used as selection criteria for identifying superior drought tolerant NILs from the phenomics platform. Furthermore, considering the individual traits, PSA and NIR could be directly used for selection. Based on this criterion, a final set of nine NILs such as P1823-12-23, P1823-12-32, P1823-12-42, P1823-12-48, P1823-12-64, P1823-12-65, P1823-12-104, P1823-12-124, and P1823-12-141 were selected. These outstanding NILs performing better under both phenomics-based screening and field screening could be used for varietal development.

The pattern of stage-wise correlation of phenomics parameters with yield provided another clue. It was found that a high positive correlation with grain yield got stronger at stage III of the phenomics screening, particularly for the PSA. Stage III had the peak drought, following which the plant has entered into the recovery phase after lifesaving irrigation. Therefore, it must be understood that the best correlation with the grain yield under drought was obtained by the phenomics parameters recorded at the peak drought stage. The strength of correlation was found to go weak at the recovery phase. This gave us the indication of the criticality of the stage at which comparison is to be made for selection purposes. At stage I, wherein no statistically validated differences were available between stressed and unstressed treatments, the association of the PSA with yield was weak, which may be due to the lack of apparent drought response at this stage. As the drought started to show its effect on PSA beginning from stage II, the correlation got stronger and reached its maximum at stage III when the drought was at its peak. At the recovery phase, the strength of association began to revert to a lower magnitude. This implied that the drought response such as leaf rolling and wilting could predict the drought tolerance response among the NILs. However, in this study, we did not have enough data proving this hypothesis but it is worth examining in a large NIL set with several plants. From the observations herein, we suggest that the phenomics parameters at peak drought stage (stage III in this case) could be considered critical for the selection of drought-tolerant lines.

Several drought indices were used in previous works targeted to identify drought-tolerant lines/genotypes in many crops (Babu et al., 2011; Dadbakhsh et al., 2011; Farshadfar and Elyasi, 2012; Naghavi et al., 2013; Ali and El-Sadek, 2016; Bennani et al., 2017; Mau et al., 2019), since a simultaneous selection for yield and tolerance indices could be a good selection criterion (Raman et al., 2012; Garg and Bhattacharya, 2017; Oo et al., 2021a). As different indices were having separate response emphasis, it would be prudent to use multiple indices for a comprehensive evaluation of drought tolerance (Oo et al., 2021a). Because of this reason, we have used multiple drought indices in the present study and found that indices such as TOL and SSI showed a good correlation with image-based parameters. This provided a further implication of the use of tolerant indices along with phenomics parameters and yield. No previous study comparing the drought indices and phenomics parameters has been reported earlier. Based on the field performance, we could select a final set of nine NILs. These NILs can be further evaluated for national testing for cultivar release and can be used as parental lines for drought tolerance breeding.

## Conclusion

A set of 35 NILs of Pusa 44 was simultaneously evaluated for drought response in a field evaluation as well as in a phenomics platform under induced drought. The field tolerance of the lines was confirmed from two years of evaluation and compared with the image-based phenomics traits such as PSA, WU, NIR, and TR. From the significant association of these traits, particularly PSA at peak drought, we found that phenomics-based traits can be directly used as selection criteria for identifying drought-tolerant lines. In addition, the phenomics evaluation provided an early-stage non-invasive method that can be carried on continuously and scaled to a large population, and automated. This opens a novel vista in the evaluation and selection of rice breeding lines based on various physiological parameters. Additionally, if scaled up, this method could be labor-saving, cost-effective, and accurate as well as could overcome the limitations of conventional drought screening methods such as using rainout shelters. Rapid and high throughput phenotyping is the need of the hour, for scaling up the breeding programs to genomic selection pipelines as well as in accelerating genetic gain. Besides, accelerated approaches provide promise to generate climate-resilient cultivars to ensure future food security, especially under the changing climate and water limitations.

## Data Availability Statement

The original contributions presented in the study are included in the article/Supplementary Material, further inquiries can be directed to the corresponding author.

## Author Contributions

SG and VC: conceptualization. SG, VC, MP, and DR: methodology. KV, PB, RE, and SG: software. PD and GD: validation. PD, GD, KV, and SG: formal analysis. PD, PB, HB, SG, KV, VC, and DR: investigation. SG, AS, MP, MN, and DR: resources. PD, KV, and SG: data curation, visualization, and writing – original draft preparation. KV and SG: writing – review and editing. SG, AS, NR, VC, and DR: supervision. SG, MP, and AS: project administration. SG, MP, and VC: funding acquisition. All authors have read and agreed to the published version of the manuscript.

## Conflict of Interest

The authors declare that the research was conducted in the absence of any commercial or financial relationships that could be construed as a potential conflict of interest.

## Publisher’s Note

All claims expressed in this article are solely those of the authors and do not necessarily represent those of their affiliated organizations, or those of the publisher, the editors and the reviewers. Any product that may be evaluated in this article, or claim that may be made by its manufacturer, is not guaranteed or endorsed by the publisher.

## Footnotes

1
www.irri.org/climate-change-ready-rice
