# Supplementary material for: Drought Tolerant Near Isogenic Lines of Pusa 44 Pyramided With qDTY2.1 and qDTY3.1, Show Accelerated Recovery Response in a High Throughput Phenomics Based Phenotyping
Source: Front Plant Sci. 2022 Jan 5;12:752730. doi: 10.3389/fpls.2021.752730 (PMC8767905; doi:10.3389/fpls.2021.752730)

Frontiers in Plant Science 12:752730
doi: 10.3389/fpls.2021.752730

Supplementary information

Drought tolerant near isogenic lines of Pusa 44 pyramided with *qDTY2.1* and *qDTY3.1*, shows accelerated recovery response in a high throughput phenomics based phenotyping

Priyanka Dwivedi, Naleeni Ramawat, Dhandapani Raju, Gaurav Dhawan, S Gopala Krishnan, Viswanathan Chinnusamy, Prolay Kumar Bhowmick, KK Vinod, Madan Pal, M Nagarajan, Ranjith Kumar Ellur, Haritha Bollinedi, AK Singh

Supplementary Table S1. Weather data for reproductive phase during *Kharif* 16 and *Kharif* 17 for field experiment

Date	2016 field				2017 field				2016 pot			
	**Tmax °C**	**Tmin °C**	**Tmean °C**	**Rainfall mm**	**Tmax °C**	**Tmin °C**	**Tmean °C**	**Rainfall mm**	**Tmax °C**	**Tmin °C**	**Tmean °C**	**RH%**
01-Oct-16	35.0	23.0	29.0	0.0	35.6	19.5	27.6	0	35.6	20.5	26.8	54.5
02-Oct-16	36.2	23.5	29.9	0.0	35.5	19.4	27.5	0	32.9	21.8	27.7	55.9
03-Oct-16	35.0	24.0	29.5	0.0	35.6	18.8	27.2	0	32.8	21.9	27.7	55.4
04-Oct-16	36.2	24.2	30.2	0	34.8	19.8	27.3	0	33.3	21.0	27.8	53.6
05-Oct-16	35.0	21.4	28.2	37.8	35.4	21.0	28.2	0	33.2	21.5	27.1	55.5
06-Oct-16	31.0	22.0	26.5	0.0	35.5	20.6	28.1	0	32.8	21.7	27.1	54.7
07-Oct-16	33.0	21.0	27.0	0.0	35.0	18.4	26.7	0	36.9	21.4	27.1	55.5
08-Oct-16	34.2	19.4	26.8	0.0	34.0	19.6	26.8	0	33.0	22.0	27.3	55.4
09-Oct-16	35.0	18.8	26.9	0.0	34.0	19.4	26.7	0	32.8	21.3	27.1	55.0
10-Oct-16	34.8	20.2	27.5	0.0	34.5	19.5	27.0	0	33.4	21.9	27.3	55.5
11-Oct-16	33.1	16.7	24.9	0.0	34.0	20.0	27.0	0	33.6	21.9	27.4	57.3
12-Oct-16	34.0	16.0	25.0	0.0	34.0	19.6	26.8	0	33.2	22.0	27.2	57.4
13-Oct-16	34.5	14.4	24.5	0.0	34.3	19.2	26.8	0	40.0	21.9	27.9	57.6
14-Oct-16	34.8	14.5	24.7	0.0	35.0	17.6	25.3	0	32.8	21.8	27.3	57.4
15-Oct-16	35.0	13.5	24.5	0.0	35.5	16.2	25.9	0	32.7	21.8	27.1	56.9
16-Oct-16	33.5	13.2	23.4	0.0	35.8	15.6	25.7	0	32.6	21.9	27.1	56.8
17-Oct-16	34.0	13.0	23.5	0.0	34.8	16.0	25.4	0				
18-Oct-16	34.0	13.4	23.7	0.0	34.0	16.6	25.3	0				
19-Oct-16	34.0	13.4	23.7	0.0	34.4	16.8	25.6	0				
20-Oct-16	34.3	13.6	24.0	0.0	35.2	17.4	26.3	0				
21-Oct-16	35.0	14.8	24.9	0.0	34.6	15.9	25.3	0				
22-Oct-16	34.2	14.6	24.4	0.0	34.6	15.2	24.9	0				
23-Oct-16	35.0	14.5	24.8	0.0	34.6	15.7	25.2	0				
24-Oct-16	32.5	13.6	23.1	0.0	34.2	14.6	24.4	0				
25-Oct-16	34.0	14.8	24.4	0.0	32.8	14.0	23.4	0				
26-Oct-16	32.4	13.7	23.1	0.0	31.5	13.8	22.7	0				
27-Oct-16	33.6	11.6	22.7	0.0	32.2	16.0	24.1	0				
28-Oct-16	32.5	11.6	22.1	0.0	30.6	14.2	22.4	0				
29-Oct-16	31.8	10.6	21.2	0.0	31.2	14.8	23.0	0				
30-Oct-16	32.4	11.0	21.7	0.0	30.2	14.5	22.4	0				
31-Oct-16	30.8	11.8	21.3	0.0	29.8	14.5	22.2	0				
Total	**1050.8**	**501.8**	**777.1**	**37.8**	**1053.2**	**534.2**	**793.2**	**0.0**				
Mean	**33.9**	**16.2**	**25.1**	**1.2**	**34.0**	**17.2**	**25.6**	**0.0**				

No. of lifesaving irrigations – Kharif 16 (pot experiment) - One
Kharif 16 (Field experiment) - One
Kharif 17 (Field experiment) – Two

Supplementary Table S2. Variation for phenomic parameters under drought stressed and unstressed conditions of controlled environment phenotyping

Traits	Stages	Unstressed (irrigated)					Stressed (drought)					F-value		
		**P1**	**P2**	**NILs**	**Mean**	**CD (0.05)**	**P1**	**P2**	**NILs**	**Mean**	**CD (0.05)**	**Genotype**	**Treatment**	**G*T**
PSA	Stage I	331.6	461.8	374.8	376.0	14.0	320.24	501.1	370.6	370.6	20.8	2.2	0.3	21.4**
	Stage II	370.8	368.6	378.3	380.6	15.5	266.63	269.0	371.6	372.3	20.5	2.3	0.7	
	Stage III	379.1	392.5	359.2	362.0	14.4	300.89	329.8	278.2	277.6	13.3	2.5*	132.2*	
	Stage IV	327.7	513.2	353.1	355.8	13.4	306.40	472.7	303.0	303.7	15.1	2.5*	48.3*	
	Stage V	281.1	281.3	342.8	345.4	13.9	300.28	339.0	309.5	310.1	15.6	2.2*	18.8*	
	Mean	338.1	403.5	361.6	-	-	298.89	382.3	326.6	-	-	-	-	
	CV%	12.0	22.5	4.6	-	-	6.8	26.5	13.4	-	-	-	-	
WU	Stage I	6.5	7.6	6.2	6.3	0.3	7.9	7.1	7.0	7.1	0.4	1.9	14.9	7.0**
	Stage II	6.4	7.5	5.7	5.7	0.3	5.6	5.1	4.6	4.6	0.3	1.1	32.6*	
	Stage III	6.7	7.4	6.4	6.4	0.3	2.7	2.2	2.6	2.6	0.2	1.3	459.2*	
	Stage IV	6.7	7.9	6.0	6.0	0.4	6.0	4.9	5.2	5.2	0.4	2.0*	16.2*	
	Stage V	6.9	7.8	6.6	6.6	0.4	6.6	6.1	6.1	6.1	0.6	2.0*	3.6*	
	Mean	6.6	7.6	6.2	-	-	5.7	5.1	5.1	-	-	-	-	
	CV%	3.3	3.0	6.5	-	-	34.2	36.8	33.7	-	-	-	-	
TR	Stage I	0.07	0.07	0.07	0.07	0.00	0.07	0.07	0.07	0.07	0.00	0.9	0	3.9**
	Stage II	0.07	0.06	0.07	0.07	0.00	0.05	0.05	0.05	0.05	0.00	1.1	180.2*	
	Stage III	0.08	0.06	0.08	0.08	0.00	0.03	0.03	0.04	0.04	0.00	1.4	638.3*	
	Stage IV	0.08	0.06	0.08	0.08	0.00	0.05	0.05	0.06	0.06	0.00	2.8*	52.8*	
	Stage V	0.08	0.06	0.08	0.08	0.00	0.05	0.06	0.06	0.06	0.00	2.1*	94.0*	
	Mean	0.08	0.06	0.07	-	-	0.05	0.05	0.06	-	-	-	-	
	CV%	7.5	7.7	7.9	-	-	28.6	30.4	19.6	-	-	-	-	
NIR	Stage I	166.0	176.0	167.0	167.0	1.0	165.0	174.0	167.0	167.0	1.0	1. 7	0.2	6.8**
	Stage II	168.0	177.0	169.0	169.0	1.1	176.0	181.0	173.0	174.0	1.3	1.7	38.1*	
	Stage III	168.0	171.0	173.0	172.0	1.3	192.0	191.0	185.0	186.0	1.7	1.3	184.4*	
	Stage IV	170.0	171.0	173.0	173.0	1.4	183.0	173.0	178.0	178.0	1.4	1.5*	31.1*	
	Stage V	177.0	179.0	181.0	181.0	1.3	184.0	179.0	180.0	181.0	0.8	0.9	0.1	
	Mean	170.0	175.0	172.0	-	-	180.0	179.0	177.0	177.0	-	-	-	
	CV%	3.5	2.5	3.6	-	-	6.0	4.8	4.6	-	-	-	-	

P1, Pusa 44; P2, IR 81896-B-B-142; PSA, projected shoot area in cm^2; WU, water use in ml/g/day; TR, transpiration rate in g per cm^2; NIR, near infrared value; CD, critical difference; * significant at 5%; Stage I, II, III, IV and V indicates different stages of stress and recovery; CV, coefficient of variation; G, genotype; T, treatment.

Supplementary Table S3. Mean performance of Pusa 44 NILs for projected shoot area (PSA) expressed in cm^2 under stress and unstressed treatments in the controlled environment phenotyping. The means under each treatment, followed by same letters are statistically not significant at 5% level by least significance difference test.

ENTRY	Projected shoot area (cm^2)									
	Stage I		Stage II		Stage III		Stage IV		Stage V	
	Unstress	Stress	Unstress	Stress	Unstress	Stress	Unstress	Stress	Unstress	Stress
P1823-12-4	339no	374^{jk}	337.3^{o-q}	376.5^{h-k}	321^{m-o}	270^{fgh}	312.3^{m-o}	227.1^{q}	298^{o-r}	291^{klm}
P1823-12-21	429^{bc}	407^{e-h}	447.7^{c}	406.3^{d-f}	421^{c}	369^{a}	409.5^{b}	403.1^{a}	398^{cd}	379^{b}
P1823-12-23	427^{bc}	462^{b-d}	435.0^{cd}	474.7^{ab}	419^{cd}	313^{b}	405.7^{b}	307.5^{ij}	397^{cd}	369^{b}
P1823-12-32	395^{fg}	383^{ij}	398.4^{f-h}	391.1^{f-i}	381^{ef}	275^{efg}	370.4^{c-e}	316.9^{hi}	353^{f}	332^{c-f}
P1823-12-36	352^{mn}	342^{m-o}	361.6^{j-m}	348.8^{lm}	322^{m-o}	247^{jkl}	306.6^{no}	255.6^{m-o}	288^{r}	274^{n-q}
P1823-12-42	420^{cd}	382^{ij}	404.2^{fg}	397.7^{e-g}	379^{ef}	287^{e}	372.4^{cd}	328.4^{f-h}	356^{f}	345^{c}
P1823-12-44	357^{lm}	364^{j-l}	348.9^{m-o}	367.6^{j-l}	337^{j-l}	273^{efg}	353.3^{f-i}	319.7^{g-i}	342^{g-i}	300^{j-l}
P1823-12-48	373^{h-k}	364^{j-l}	397.0^{f-h}	372.7^{h-k}	378^{e-g}	268^{ghi}	358.1^{e-h}	334.9^{fg}	335^{i-k}	329^{c-g}
P1823-12-49	370 $^{i-l}$	322^{op}	383.6^{hi}	324.9^{no}	377^{e-g}	245j-^{m}	368.1^{c-e}	239.8^{pq}	350^{f-h}	287^{l-n}
P1823-12-50	397^{fg}	396^{e-i}	409.5^{f}	408.6^{d-f}	384^{ef}	333^{c}	374.2^{c}	325.4^{f-h}	359^{f}	404^{a}
P1823-12-63	462^{a}	370^{jk}	468.8^{b}	370.2^{i-k}	418^{cd}	277^{efg}	404.8^{b}	278.8^{k}	392^{de}	313^{h-j}
P1823-12-64	314^{q}	346^{l-n}	321.7^{q-s}	360.5^{i-m}	316no	247^{jkl}	308.6^{m-o}	252.1^{n-p}	292^{r}	303^{i-k}
P1823-12-65	339no	361^{k-m}	340.0^{n-p}	359.8^{i-m}	311^{op}	243^{klm}	301.1^{o}	268.3^{k-m}	293^{qr}	318^{f-i}
P1823-12-77	357^{lm}	370^{jk}	343.5^{n-p}	389.1^{f-i}	334^{k-m}	271^{fgh}	333.1^{kl}	262.5^{l-o}	330^{i-l}	337 $^{c-e}$
P1823-12-79	377^{h-j}	288^{rs}	377.1^{ij}	286.2^{pq}	340^{j-l}	227^{n}	353.8^{f-i}	270.9^{kl}	362^{f}	222 ^{t}
P1823-12-80	430^{bc}	336no	433.3^{cd}	342.2^{mn}	405^{d}	263^{ghi}	412.0^{b}	300.2^{j}	418^{b}	26 $^{o-r}$
P1823-12-81	324^{pq}	297^{qr}	332.3^{p-r}	301.2^{p}	329 $^{l-n}$	244^{klm}	317.3^{mn}	250.7^{op}	297^{p-r}	259^{qr}
P1823-12-82	427^{bc}	444^{d}	426.0^{de}	462.0^{ab}	391^{e}	344^{bc}	371.4^{c-e}	334.0^{fg}	352^{fg}	371^{b}
P1823-12-84	358^{lm}	417^{e}	361.1^{k-m}	425.9^{cd}	337 $^{j-l}$	305^{d}	348.3^{g-j}	267.2^{k-n}	327^{j-l}	327^{d-h}
P1823-12-89	332^{op}	260^{t}	342.3^{n-p}	256.6^{r}	330 $^{l-n}$	236^{lmn}	348.1^{g-j}	275.4^{kl}	331^{i-l}	238^{s}
P1823-12-96	333^{op}	272st	354.8^{l-n}	270.2^{qr}	347^{i-k}	239^{lmn}	345.2^{h-k}	209.4^{r}	337^{h-j}	254^{r}
P1823-12-98	386^{gh}	297^{qr}	388.6^{g-i}	301.7^{p}	377^{e-g}	240^{lmn}	362.9^{c-f}	300.9 ^{j}	352^{fg}	276^{m-p}
P1823-12-104	321^{pq}	288^{rs}	317.0^{rs}	277.0^{qr}	309^{op}	199^{o}	321.2^{lm}	279.0^{k}	311^{m-o}	219^{t}
P1823-12-114	362^{k-m}	342^{m-o}	365.2^{j-l}	355.2^{k-m}	349^{ij}	258^{hij}	339.6^{jk}	335.7^{ef}	324^{k-m}	330^{c-g}
P1823-12-118	437^{b}	411^{ef}	448.7^{c}	412.1^{de}	443^{b}	304^{d}	412.0^{b}	391.7^{ab}	409^{bc}	324^{d-h}
P1823-12-120	335^{op}	341^{mno}	326.5^{p-r}	325.9^{no}	298^{p}	255^{ijk}	283.6^{p}	318.3^{hi}	265^{s}	267^{p-r}
P1823-12-122	397^{fg}	481^{a}	409.5^{f}	459.7^{b}	384^{ef}	330^{c}	374.2^{c}	272.5^{kl}	359^{f}	363^{b}
P1823-12-123	403^{ef}	486^{a}	401.4^{fg}	481.6 ^{a}	380 ^{ef}	351 ^{b}	370.8 $^{c-e}$	329.7 $^{f-h}$	363 ^{f}	410 ^{a}

ENTRY	Projected shoot area (cm^2)									
	Stage I		Stage II		Stage III		Stage IV		Stage V	
	Unstress	Stress	Unstress	Stress	Unstress	Stress	Unstress	Stress	Unstress	Stress
P1823-12-124	327^{o-q}	456^{cd}	334.7^{o-q}	438.1^{c}	341^{j-l}	302^{d}	333.7^{kl}	357.5^{d}	320^{l-n}	322^{e-h}
P1823-12-127	412^{de}	311^{pq}	411.4^{ef}	314.6^{op}	374^{fg}	255^{ijk}	362.0^{c-f}	299.1^{j}	353^{f}	290^{k-l}
P1823-12-130	393^{fg}	484^{a}	397.5^{f-h}	460.3^{b}	364^{gh}	333^{c}	344.3^{i-k}	364.5^{cd}	339^{g-j}	315^{g-j}
P1823-12-132	383^{g-i}	473^{ab}	376.7^{i-k}	460.7^{b}	359^{hi}	343^{bc}	371.7^{cd}	379.4^{bc}	381^{e}	366^{b}
P1823-12-134	368^{j-l}	408^{efg}	366.1^{j-l}	408.8^{d-f}	357^{hi}	283^{ef}	346.4^{h-k}	350.2^{de}	360^{f}	284^{m-o}
P1823-12-141	365^{j-m}	335no	364.9^{j-l}	309.2^{op}	359^{hi}	233^{mn}	359.9^{d-g}	298.4^{j}	350^{f-h}	281^{m-p}
P1823-12-143	313^{q}	303^{pqr}	306.5^{s}	306.4^{op}	300^{p}	274^{efg}	300.7^{o}	322.3^{f-i}	307^{n-p}	268^{pqr}
Pusa 44	332^{op}	371^{jk}	333.8^{o-q}	379.1^{g-j}	320^{m-o}	267^{ghi}	318.1^{mn}	280.0^{k}	306^{n-q}	300^{jkl}
IR81896-B-B-142	462^{a}	369^{jk}	510.7^{a}	392.5^{e-h}	501^{a}	269^{fgh}	490.1^{a}	328.3^{f-h}	473^{a}	339^{cd}
SED	6.88	10.25	7.666	10.122	7.1	6.56	6.593	7.459	6.83	7.71
CD (0.05)	13.95	20.78	15.548	20.529	14.41	13.31	13.371	15.127	13.85	15.63

Supplementary Table S4. Mean performance of Pusa 44 NILs for water use (WU) expressed in ml/g/day under stress and unstressed treatments in the controlled environment phenotyping. The means under each treatment, followed by same letters are statistically not significant at 5% level by least significance difference test.

ENTRY	Water use (mg/ml/day)									
	Stage I		Stage II		Stage III		Stage IV		Stage V	
	Unstress	Stress	Unstress	Stress	Unstress	Stress	Unstress	Stress	Unstress	Stress
P1823-12-4	5.205^{no}	5.483^{rs}	4.494^{lm}	3.764^{q}	5.119^{st}	$1.718^{s\text{-}u}$	4.778^{o}	3.273^{w}	5.973^{no}	3.764^{pq}
P1823-12-21	6.375^{ijk}	5.319^{st}	$5.445^{i\text{-}k}$	4.468^{kl}	$6.375^{j\text{-}m}$	2.979^{ef}	$6.175^{h\text{-}k}$	$4.468^{n\text{-}q}$	7.57^{cde}	5.319^{jkl}
P1823-12-23	5.243^{no}	$6.489^{n\text{-}p}$	5.306^{jk}	$4.681^{h\text{-}k}$	5.337^{rs}	1.596^{u}	5.056^{no}	4.043^{stu}	6.18^{mn}	5.532^{ijk}
P1823-12-32	5.23^{no}	$7.002^{k\text{-}m}$	5.788^{gh}	$4.668^{i\text{-}k}$	$6.276^{k\text{-}m}$	1.966^{pqr}	6.067^{jk}	4.914^{klm}	$6.276^{k\text{-}n}$	6.634^{efg}
P1823-12-36	4.469^{p}	6.043^{qr}	3.972^{o}	4.094^{op}	3.821^{v}	1.657^{tu}	3.368^{q}	3.899^{tuv}	4.627^{st}	4.094^{op}
P1823-12-42	7.702^{ab}	$7.52^{f\text{-}i}$	6.52^{cd}	5.541^{bc}	$7.579^{b\text{-}d}$	2.639^{ijk}	6.601^{ef}	5.541^{hi}	7.335^{def}	7.124^{def}
P1823-12-44	7.143^{cde}	$7.775^{e\text{-}h}$	5.152^{k}	5.094^{def}	$6.206^{k\text{-}n}$	3.485^{bc}	5.855^{kl}	5.63^{gh}	7.26^{efg}	7.775^{c}
P1823-12-48	6.366^{ijk}	7.975^{de}	5.681^{ghi}	5.521^{bc}	6.776^{gh}	3.067^{e}	6.16^{ijk}	6.442^{c}	$7.392^{c\text{-}f}$	8.896^{b}
P1823-12-49	5.936^{m}	5.852^{r}	5.433^{ijk}	4.107^{op}	$6.439^{i\text{-}m}$	$2.669^{h\text{-}k}$	$6.237^{g\text{-}j}$	5.133^{jkl}	7.646^{cd}	6.982^{ef}
P1823-12-50	6.792^{fg}	8.255^{cd}	5.933^{fg}	5.296^{cd}	7.026^{fg}	3.583^{b}	6.557^{efg}	7.165^{b}	8.197^{b}	9.657^{a}
P1823-12-63	6.381^{ijk}	5.908^{r}	5.182^{k}	4.376^{lm}	$6.148^{l\text{-}o}$	3.282^{d}	6.032^{jk}	5.908^{efg}	$6.265^{k\text{-}n}$	6.565^{fg}
P1823-12-64	5.403^{n}	7.568^{fghi}	5.72^{ghi}	5.459^{bc}	$6.462^{h\text{-}l}$	$2.854^{e\text{-}h}$	6.78^{cde}	6.203^{cde}	$7.415^{c\text{-}f}$	7.196^{de}
P1823-12-65	6.733^{fgh}	7.234^{ijk}	6.475^{cd}	$4.822^{g\text{-}j}$	6.954^{fg}	$2.792^{f\text{-}i}$	7.064^{c}	6.853^{b}	7.506^{cde}	8.122^{c}
P1823-12-77	6.024^{m}	7.09^{klm}	4.552^{lm}	5.134^{de}	5.522^{qr}	3.301^{cd}	5.02^{no}	6.112^{cde}	5.622^{op}	7.579^{cd}
P1823-12-79	6.044^{lm}	7.031^{klm}	4.689^{l}	4.241^{lmo}	5.385^{rs}	2.567^{jkl}	5.275^{mn}	$4.464^{n\text{-}q}$	5.934^{no}	4.688^{mn}
P1823-12-80	7.657^{ab}	7.483^{hij}	5.711^{ghi}	4.989^{efg}	$7.632^{a\text{-}d}$	$2.721^{g\text{-}j}$	$6.542^{e\text{-}h}$	4.762^{mn}	$6.542^{i\text{-}l}$	4.762^{lmn}
P1823-12-81	5.288^{no}	5.872^{r}	4.576^{lm}	3.67^{qr}	5.844^{opq}	2.477^{klm}	5.566^{lm}	$4.587^{m\text{-}p}$	6.679^{ij}	$5.688^{h\text{-}k}$
P1823-12-82	5.287^{no}	9.673^{a}	4.002^{no}	6.548^{a}	4.928^{tu}	3.869^{a}	4.839^{o}	7.738^{a}	5.376^{pq}	9.922^{a}
P1823-12-84	5.433^{n}	6.703^{mno}	4.359^{m}	4.615^{jk}	5.249^{rst}	2.198^{no}	4.236^{p}	$4.396^{o\text{-}s}$	4.788^{s}	5.934^{hi}
P1823-12-89	5.041^{o}	4.868^{u}	5.556^{hij}	3.448^{r}	$6.379^{j\text{-}m}$	2.535^{jkl}	$6.379^{f\text{-}j}$	$4.26^{p\text{-}s}$	6.79^{hi}	4.462^{no}
P1823-12-96	$6.195^{j\text{-}m}$	4.95^{tu}	6.858^{b}	3.762^{q}	7.412^{de}	2.376^{lmn}	7.08^{c}	4.752^{mno}	7.301^{def}	4.158^{nop}
P1823-12-98	6.848^{efg}	$6.736^{l\text{-}o}$	6.632^{bc}	4.922^{efgh}	7.494^{bcde}	2.979^{ef}	7.494^{b}	6.218^{cde}	7.494^{cde}	6.995^{ef}
P1823-12-104	7.629^{ab}	$6.848^{k\text{-}n}$	6.573^{bcd}	3.478^{r}	$7.629^{a\text{-}d}$	1.957^{pqr}	7.512^{b}	4.783^{lmn}	8.685^{a}	5.652^{hijk}
P1823-12-114	6.629^{ghi}	6.897^{klm}	6.142^{ef}	5.305^{cd}	$6.404^{i\text{-}m}$	3.448^{bcd}	5.618^{lm}	6.366^{cd}	5.169^{qr}	7.162^{de}
P1823-12-118	6.111^{klm}	5.464^{rs}	6.444^{cde}	3.643^{qr}	7.222^{ef}	1.821^{rst}	6.889^{cde}	3.643^{v}	7.111^{fgh}	4.736^{mn}
P1823-12-120	5.239^{no}	6.223^{pqr}	4.674^{lm}	3.863^{pq}	4.622^{u}	2.253^{n}	4.006^{p}	3.863^{uv}	4.544^{st}	3.648^{pq}
P1823-12-122	7.256^{cd}	$7.629^{e\text{-}h}$	7.407^{a}	4.46^{kl}	7.823^{ab}	2.113^{nop}	7.029^{cd}	$4.225^{q\text{-}t}$	6.803^{hi}	3.991^{op}
P1823-12-123	7.447^{bc}	8.75^{b}	7.407^{a}	5.556^{b}	7.92^{a}	2.917^{efg}	7.565^{b}	6.389^{cd}	7.329^{def}	6.944^{ef}

ENTRY	Water use (mg/ml/day)									
	Stage I		Stage II		Stage III		Stage IV		Stage V	
	**Unstress**	**Stress**	**Unstress**	**Stress**	**Unstress**	**Stress**	**Unstress**	**Stress**	**Unstress**	**Stress**
P1823-12-124	6.334^{i-l}	8.673^{b}	6.27^{de}	5.102^{def}	6.62^{hij}	2.296^{mn}	6.526^{e-i}	5.357^{hij}	6.526^{I-m}	5.867^{hij}
P1823-12-127	5.164^{no}	6.711^{mno}	7.28^{a}	4.027^{op}	7.746^{abc}	2.573^{jk}	7.981^{a}	6.04^{def}	8.92^{a}	8.054^{c}
P1823-12-130	7.002^{def}	8.437^{bc}	5.74^{ghi}	4.715^{hij}	6.535^{h-k}	2.481^{klm}	6.017^{jk}	5.211^{ijk}	6.639^{ijk}	6.948^{ef}
P1823-12-132	6.993^{def}	6.404^{opq}	6.294^{de}	3.484^{r}	5.758^{pq}	2.049^{opq}	4.196^{p}	4.098^{r-u}	4.895^{rs}	5.533^{ijk}
P1823-12-134	7.787^{a}	7.827^{efg}	4.44^{lm}	4.206^{mo}	5.533^{qr}	1.869^{qrs}	5.123^{no}	4.439^{n-r}	6.352^{j-m}	3.271^{q}
P1823-12-141	6.41^{ijk}	8.676^{b}	5.85^{fgh}	4.118^{o}	6.312^{j-m}	2.206^{no}	6.114^{jk}	4.147^{q-u}	5.917^{no}	3.235^{q}
P1823-12-143	4.989^{op}	8.596^{bc}	5.54^{hij}	4.871^{fghi}	5.876^{nop}	3.438^{bcd}	4.878^{o}	5.731^{fgh}	4.435^{t}	5.158^{klm}
Pusa 44	6.455^{hij}	7.891^{def}	6.38^{cde}	5.587^{b}	6.729^{ghi}	2.654^{ijk}	6.674^{def}	6.006^{ef}	6.893^{ghi}	6.564^{fg}
IR81896-B-B-142	7.59^{ab}	7.097^{jkl}	7.47^{a}	5.085^{def}	7.431^{cde}	2.225^{no}	7.856^{ab}	4.873^{klm}	7.749^{c}	6.144^{gh}
SED	0.152	0.191	0.16	0.121	0.163	0.096	0.186	0.176	0.186	0.282
CD (0.05)	0.308	0.388	0.324	0.246	0.33	0.196	0.376	0.357	0.378	0.572

Supplementary Table S5. Mean performance of Pusa 44 NILs for transpiration rate (TR) expressed in g/cm^2 under stress and unstressed treatments in the controlled environment phenotyping. The means under each treatment, followed by same letters are statistically not significant at 5% level by least significance difference test.

ENTRY	Transpiration rate (g/cm^2)									
	Stage I		Stage II		Stage III		Stage IV		Stage V	
	Unstress	Stress	Unstress	Stress	Unstress	Stress	Unstress	Stress	Unstress	Stress
P1823-12-4	0.069^{ijk}	0.075^{b}	0.062^{jk}	0.051^{de}	0.072^{jkl}	0.032^{jk}	0.069^{jk}	0.056^{l}	0.072^{ij}	0.057^{j}
P1823-12-21	0.075^{de}	0.068^{h}	0.065^{hi}	0.057^{a}	0.076^{hi}	0.042^{c}	0.076^{gh}	0.061^{ij}	0.078^{efg}	0.062^{hi}
P1823-12-23	0.066^{l}	0.073^{cd}	0.067^{gh}	0.051^{de}	0.068^{mn}	0.027^{mn}	0.067^{kl}	0.055^{l}	0.068^{klm}	0.054^{k}
P1823-12-32	0.074^{def}	0.074^{bc}	0.078^{a}	0.049^{fg}	0.086^{cd}	0.029^{lm}	0.086^{bc}	0.063^{hij}	0.08^{de}	0.063^{gh}
P1823-12-36	0.067^{kl}	0.07^{fg}	0.066^{hi}	0.046^{i}	0.065^{no}	0.027^{mn}	0.061^{no}	0.06^{jk}	0.064^{n}	0.06^{i}
P1823-12-42	0.072^{fgh}	0.075^{b}	0.064^{ij}	0.053^{bc}	0.077^{gh}	0.035^{ghi}	0.072^{ij}	0.065^{fgh}	0.078^{efg}	0.061^{hi}
P1823-12-44	0.07^{hij}	0.07^{fg}	0.062^{jk}	0.052^{cd}	0.076^{hi}	0.048^{ab}	0.074^{hi}	0.07^{de}	0.082^{cde}	0.07^{bc}
P1823-12-48	0.069^{ijk}	0.071^{ef}	0.06^{kl}	0.048^{gh}	0.073^{ijk}	0.037^{efg}	0.07^{jk}	0.068^{ef}	0.075^{ghi}	0.066^{ef}
P1823-12-49	0.066^{l}	0.068^{h}	0.059^{lm}	0.047^{hi}	0.071^{klm}	0.041^{cd}	0.07^{jk}	0.069^{de}	0.074^{hi}	0.067^{def}
P1823-12-50	0.072^{fgh}	0.074^{bc}	0.065^{hi}	0.046^{i}	0.08^{fg}	0.038^{def}	0.077^{fgh}	0.065^{fgh}	0.08^{de}	0.063^{gh}
P1823-12-63	0.069^{ijk}	0.066^{i}	0.066^{hi}	0.049^{fg}	0.08^{fg}	0.049^{a}	0.08^{ef}	0.07^{de}	0.083^{cd}	0.06^{i}
P1823-12-64	0.062^{m}	0.073^{cd}	0.067^{gh}	0.051^{de}	0.076^{hi}	0.039^{de}	0.082^{de}	0.075^{bc}	0.084^{c}	0.069^{cd}
P1823-12-65	0.069^{ijk}	0.066^{i}	0.07^{ef}	0.05^{ef}	0.078^{fgh}	0.038^{def}	0.082^{de}	0.075^{bc}	0.084^{c}	0.071^{bc}
P1823-12-77	0.071^{ghi}	0.065^{i}	0.059^{lm}	0.05^{ef}	0.074^{hij}	0.042^{c}	0.069^{jk}	0.065^{fgh}	0.071^{ijk}	0.062^{hi}
P1823-12-79	0.07^{hij}	0.07^{fg}	0.075^{bc}	0.051^{de}	0.09^{abc}	0.039^{de}	0.093^{a}	0.068^{ef}	0.091^{ab}	0.069^{cd}
P1823-12-80	0.082^{ab}	0.07^{fg}	0.065^{hi}	0.054^{b}	0.07^{lm}	0.038^{def}	0.062^{mn}	0.064^{ghi}	0.066^{mn}	0.065^{fg}
P1823-12-81	0.079^{bc}	0.07^{fg}	0.071^{ef}	0.051^{de}	0.091^{ab}	0.042^{c}	0.088^{bc}	0.077^{b}	0.093^{a}	0.074^{a}
P1823-12-82	0.073^{efg}	0.073^{cd}	0.062^{jk}	0.048^{gh}	0.078^{fgh}	0.038^{def}	0.078^{fg}	0.065^{fgh}	0.082^{cde}	0.062^{hi}
P1823-12-84	0.069^{ijk}	0.073^{cd}	0.06^{kl}	0.049^{fg}	0.074^{hij}	0.033^{ij}	0.063^{mn}	0.06^{jk}	0.067^{lmn}	0.061^{hi}
P1823-12-89	0.075^{de}	0.071^{ef}	0.077^{ab}	0.051^{de}	0.088^{bc}	0.041^{cd}	0.088^{bc}	0.067^{efg}	0.09^{ab}	0.068^{cde}
P1823-12-96	0.07^{hij}	0.071^{ef}	0.074^{cd}	0.054^{b}	0.08^{fg}	0.039^{de}	0.077^{fgh}	0.072^{cd}	0.079^{ef}	0.073^{ab}
P1823-12-98	0.077^{cd}	0.073^{cd}	0.078^{a}	0.052^{cd}	0.088^{bc}	0.04^{cd}	0.089^{b}	0.076^{b}	0.093^{a}	0.073^{ab}
P1823-12-104	0.084^{a}	0.07^{fg}	0.077^{ab}	0.052^{cd}	0.091^{ab}	0.035^{ghi}	0.093^{a}	0.081^{a}	0.093^{a}	0.07^{bc}
P1823-12-114	0.072^{fgh}	0.069^{gh}	0.069^{fg}	0.051^{de}	0.076^{hi}	0.046^{b}	0.069^{jk}	0.068^{ef}	0.074^{hi}	0.066^{ef}
P1823-12-118	0.063^{m}	0.073^{cd}	0.064^{ij}	0.049^{fg}	0.073^{ijk}	0.033^{ij}	0.072^{ij}	0.054^{l}	0.076^{fgh}	0.061^{hi}
P1823-12-120	0.073^{efg}	0.071^{ef}	0.07^{ef}	0.046^{i}	0.072^{jkl}	0.034^{hij}	0.065^{lm}	0.055^{l}	0.07^{jkl}	0.056^{jk}
P1823-12-122	0.073^{efg}	0.075^{b}	0.075^{bc}	0.046^{i}	0.082^{ef}	0.03^{kl}	0.075^{ghi}	0.057^{kl}	0.079^{ef}	0.061^{hi}
P1823-12-123	0.071^{ghi}	0.072^{de}	0.074^{cd}	0.046^{i}	0.08^{fg}	0.033^{ij}	0.078^{fg}	0.063^{hij}	0.08^{de}	0.062^{hi}

ENTRY	Transpiration rate (g/cm^2)									
	Stage I		Stage II		Stage III		Stage IV		Stage V	
	**Unstress**	**Stress**	**Unstress**	**Stress**	**Unstress**	**Stress**	**Unstress**	**Stress**	**Unstress**	**Stress**
P1823-12-124	0.084^{a}	0.075^{b}	0.079^{a}	0.046^{i}	0.084^{de}	0.03^{kl}	0.085^{cd}	0.065^{fgh}	0.088^{b}	0.065^{fg}
P1823-12-127	0.07^{hij}	0.07^{fg}	0.067^{gh}	0.057^{a}	0.073^{ijk}	0.049^{a}	0.078^{fg}	0.07^{de}	0.08^{de}	0.07^{bc}
P1823-12-130	0.071^{ghi}	0.075^{b}	0.061^{kl}	0.05^{ef}	0.072^{jkl}	0.03^{kl}	0.07^{jk}	0.055^{l}	0.071^{ijk}	0.057^{j}
P1823-12-132	0.071^{ghi}	0.073^{cd}	0.051^{n}	0.041^{j}	0.063^{o}	0.032^{jk}	0.058^{o}	0.048^{m}	0.06^{o}	0.05^{l}
P1823-12-134	0.07^{hij}	0.075^{b}	0.071^{ef}	0.04^{j}	0.092^{a}	0.026^{n}	0.089^{b}	0.048^{m}	0.091^{ab}	0.05^{l}
P1823-12-141	0.074^{def}	0.073^{cd}	0.067^{gh}	0.038^{k}	0.074^{hij}	0.027^{mn}	0.072^{ij}	0.048^{m}	0.074^{hi}	0.05^{l}
P1823-12-143	0.07^{hij}	0.08^{a}	0.066^{hi}	0.046^{i}	0.072^{jkl}	0.036^{fgh}	0.065^{lm}	0.061^{ij}	0.071^{ijk}	0.062^{hi}
Pusa 44	0.071^{ghi}	0.073^{cd}	0.072^{de}	0.051^{de}	0.077^{gh}	0.034^{hij}	0.077^{fgh}	0.068^{ef}	0.08^{de}	0.05^{l}
IR81896-B-B-142	0.072^{fgh}	0.07^{fg}	0.057^{m}	0.046^{i}	0.057^{p}	0.03^{kl}	0.062^{mn}	0.054^{l}	0.065^{mn}	0.06^{i}
SED	0.001	0	0.001	0.001	0.001	0.001	0.002	0.001	0.001	0.001
CD (0.05)	0.002	0.001	0.002	0.001	0.003	0.002	0.003	0.003	0.003	0.002

Supplementary Table S6. Mean performance of Pusa 44 NILs for NIR intensity under stress and unstressed treatments in the controlled environment phenotyping. The means under each treatment, followed by same letters are statistically not significant at 5% level by least significance difference test.

ENTRY	NIR intensity									
	Stage I		Stage II		Stage III		Stage IV		Stage V	
	Unstress	Stress	Unstress	Stress	Unstress	Stress	Unstress	Stress	Unstress	Stress
P1823-12-4	162^{m}	162.6^{p}	163.8^{p}	165.3^{q}	168.6^{jkl}	174^{u}	168.6^{qrs}	167.8^{o}	180.5^{fgh}	175^{m}
P1823-12-21	165.4^{ij}	163.6^{op}	166.1^{o}	167.1^{p}	169.4^{ijk}	181.4^{p-s}	171.1^{j-m}	170.9^{n}	180.6^{fg}	177^{l}
P1823-12-23	165.8^{hi}	169.1^{e-h}	166°	171^{mn}	166.6^{mn}	180.4^{rs}	170.1^{l-q}	171.9^{mn}	179.4^{g-j}	177^{l}
P1823-12-32	171^{b}	168^{hij}	174.4^{b}	171.9^{klm}	179^{a}	185.9^{h-k}	178.5^{bc}	172.3^{mn}	189.2^{a}	178.1^{k}
P1823-12-36	166.3^{ghi}	163.3^{op}	168.4^{g-j}	167.4^{p}	172.4^{def}	180.8^{qrs}	172.8^{ghi}	174.3^{kl}	181.2^{f}	178.4^{jk}
P1823-12-42	167.6^{ef}	171.5^{bc}	167.5^{j-m}	172.5^{jkl}	167.9^{lm}	177.7^{t}	167.7^{st}	173.2^{lm}	173.8^{p}	179.4^{ghi}
P1823-12-44	166.6^{fgh}	166.2^{klm}	168.5^{g-j}	169.2^{o}	172.8^{def}	179.8^{s}	172.2^{h-k}	173.2^{lm}	179.5^{g-j}	179.5^{ghi}
P1823-12-48	165.9^{hi}	162.7^{p}	168.5^{g-j}	175.4^{fgh}	172.5^{def}	190.5^{cd}	174.3^{fg}	178^{efg}	182.9^{e}	180^{fgh}
P1823-12-49	166^{ghi}	169.1^{e-h}	166.9^{l-o}	171.8^{klm}	170.2^{hi}	176.6^{t}	169.8^{m-r}	173.8^{l}	180.7^{fg}	180.1^{fg}
P1823-12-50	169.2^{d}	168.5^{ghi}	170^{ef}	171.7^{klm}	171.7^{efg}	182.4^{n-q}	170.5^{l-o}	175.4^{jk}	178.4^{jk}	180.5^{ef}
P1823-12-63	167.7^{ef}	169.8^{de}	171.7^{d}	175.2^{gh}	179.2^{a}	186.6^{f-i}	177.3^{cde}	176.6^{g-j}	186^{c}	181^{e}
P1823-12-64	168.1^{e}	161.3^{q}	170.9^{de}	170no	172.2^{d-g}	183.1^{m-p}	172.5^{hij}	175.4^{jk}	178.7^{ijk}	180^{fgh}
P1823-12-65	169.6^{cd}	166.9^{jk}	168.7^{ghi}	174.3^{hi}	170.9^{gh}	187.8^{efg}	168.5^{rst}	176^{ij}	174.7^{op}	180^{fgh}
P1823-12-77	164.4^{jk}	168.6^{f-i}	168.2^{h-k}	172.4^{j-m}	172.3^{d-g}	177.8^{t}	176.1^{e}	176.9^{ghi}	183^{e}	182^{d}
P1823-12-79	171^{b}	168.2^{hi}	173.4^{bc}	172.7^{jk}	176.7^{b}	182.1^{o-r}	176^{e}	179^{e}	185.3^{cd}	180^{fgh}
P1823-12-80	171.3^{b}	171.6^{b}	173.9^{bc}	175.5^{fgh}	178.4^{a}	188^{efg}	182.8^{a}	178.8^{e}	185^{cd}	180^{fgh}
P1823-12-81	166.5^{ghi}	167.7^{ij}	169.9^{ef}	174.9^{hi}	173^{de}	187.6^{fgh}	172.5^{hij}	177.1^{f-i}	184.2^{de}	183^{c}
P1823-12-82	161.7^{m}	163.6^{op}	164^{p}	170.2^{no}	168.8^{jkl}	184.3^{klm}	169.8^{m-r}	176.2^{hij}	179.1^{h-k}	178.4^{jk}
P1823-12-84	169.7^{cd}	165.2^{mn}	173.2^{c}	171.2^{lmn}	178.4^{a}	183.4^{mno}	178.4^{c}	179^{e}	187.6^{b}	180^{fgh}
P1823-12-89	163.2^{l}	169.6^{def}	166^{o}	178^{cd}	169.9^{hij}	188.2^{ef}	169.5^{n-r}	177.9^{efg}	180.4^{fgh}	181^{e}
P1823-12-96	167^{efg}	168.4^{ghi}	169.4^{fg}	178.1^{c}	173.5^{d}	191.9^{bc}	174.4^{f}	181.4^{d}	179.2^{h-k}	182^{d}
P1823-12-98	166.6^{fgh}	169.4^{efg}	166.4^{mno}	173.5^{ij}	173.3^{d}	186^{h-k}	172.9^{f-i}	178.5^{ef}	178.5^{jk}	181^{e}
P1823-12-104	165.4^{ij}	172^{b}	167.1^{k-o}	181.5^{ab}	169.5^{ij}	192.2^{bc}	168.7^{p-s}	181.9^{cd}	174.9^{nop}	183^{c}
P1823-12-114	166.1^{ghi}	165.8^{lm}	169.1^{fgh}	172.9^{jk}	171.7^{efg}	184.1^{lmn}	172.2^{hijk}	178.2^{efg}	180.6^{fg}	179^{ij}
P1823-12-118	162^{m}	170.5^{cd}	162.2^{q}	176.4^{efg}	165.8^{n}	184.7^{j-m}	163.9^{u}	179^{e}	178^{kl}	181^{e}
P1823-12-120	168^{e}	167.9^{ij}	173.1^{c}	172.2^{j-m}	179^{a}	182^{o-r}	183.4^{a}	177.6^{e-h}	180^{f-i}	180^{fgh}
P1823-12-122	169.7^{cd}	163.2^{p}	170.9^{de}	172.7^{jk}	172.7^{def}	188^{efg}	172.8^{ghi}	178.8^{e}	180^{f-i}	181^{e}
P1823-12-123	170.4^{bc}	165.2^{mn}	174^{bc}	176.6^{def}	173.2^{d}	189.5^{de}	173.2^{fgh}	179^{e}	180.4^{fgh}	183^{c}

ENTRY	NIR intensity									
	**Stage I**		**Stage II**		**Stage III**		**Stage IV**		**Stage V**	
	**Unstress**	**Stress**	**Unstress**	**Stress**	**Unstress**	**Stress**	**Unstress**	**Stress**	**Unstress**	**Stress**
P1823-12-124	167.9^{e}	169.9^{de}	168.4^{g-j}	178.5^{c}	171.6^{fg}	192.4^{b}	170.9^{k-n}	182.2^{cd}	179.1^{h-k}	179.4^{ghi}
P1823-12-127	164.5^{jk}	167.8^{ij}	166.2^{no}	175.5^{fgh}	170.1^{hi}	190^{d}	172.3^{h-k}	185^{b}	181^{f}	188^{a}
P1823-12-130	163.7^{kl}	164.4^{no}	164.7^{p}	171.8^{klm}	167.4^{m}	185.3^{i-l}	167t	181.2^{d}	176.3^{mn}	180^{fgh}
P1823-12-132	166.4^{ghi}	169.8^{de}	167.6^{i-l}	180.4^{b}	169.4^{ijk}	195.6^{a}	169.2^{o-}s	183.2^{c}	175.7^{mno}	184^{b}
P1823-12-134	165.8^{hi}	165.2^{mn}	171.4^{d}	172.8^{jk}	176.8^{b}	186.3^{ghij}	177.9^{cd}	186.5^{a}	188.2^{ab}	181^{e}
P1823-12-141	171^{b}	167.9^{ij}	176^{a}	176.7^{de}	179^{a}	192.5^{b}	180^{b}	182^{cd}	180^{f-i}	182^{d}
P1823-12-143	166.2^{ghi}	166.6^{kl}	169^{fgh}	171.7^{klm}	175.2^{c}	187^{f-i}	176.5^{de}	182^{cd}	187.9^{ab}	182^{d}
Pusa 44	165.9^{hi}	165.2^{mn}	168^{h-l}	182.5^{a}	168.2^{klm}	192^{bc}	170.2^{l-p}	182.5^{cd}	176.9^{lm}	184^{b}
IR81896-B-B-142	176.5^{a}	173.5^{a}	167.4^{j-n}	180.5^{b}	171.1^{gh}	190.5^{cd}	171.4^{i-l}	173.3^{lm}	179.4^{g-j}	179.2^{hij}
SED	0.496	0.49	0.533	0.653	0.622	0.833	0.712	0.675	0.63	0.376
CD (0.05)	1.007	0.993	1.08	1.325	1.261	1.689	1.444	1.37	1.278	0.763

Supplementary Table S7. Pearson's correlation coefficient between agronomic and grain quality parameters among the NILs tested under field experimentation. Correlations of irrigated treatment are given below the diagonal, while those under the imposed drought are above diagonal. The diagonal elements in boldface indicate correlation between irrigated and drought treatments for the respective traits.

	**DFF**	**PH**	**NPT**	**PL**	**PB**	**SF%**	**YPP**	**H%**	**M%**	**TW**	**PY**	**ER**
**DFF**	**0.169**	0.577	-0.284	-0.063	-0.230	-0.148	-0.55	0.049	0.063	-0.348	-0.400	-0.110
**PH**	0.475	**0.770**	0.067	0.087	0.067	-0.241	0.064	0.138	0.171	-0.302	0.048	-0.102
**NPT**	-0.119	-0.109	**-0.199**	-0.164	0.757	0.188	0.449	-0.114	-0.114	0.067	0.229	-0.294
**PL**	0.298	0.399	0.167	**0.215**	-0.132	0.037	0.202	0.197	0.203	0.094	0.103	0.25
**PB**	0.043	0.266	0.55	0.275	**0.041**	0.059	0.49	-0.046	-0.041	0.136	0.308	-0.159
**SF%**	0.006	0.352	-0.308	0.137	0.134	**-0.164**	0.113	-0.183	-0.19	-0.077	0.083	-0.175
**YPP**	-0.064	-0.215	0.374	0.03	0.474	0.071	**0.498**	0.036	0.018	0.168	0.766	-0.111
**H%**	0.071	0.021	-0.044	0.067	0.006	0.129	0.014	**-0.284**	0.995	0.216	0.258	0.175
**M%**	-0.175	-0.082	0.101	0.005	0.198	-0.023	0.221	0.564	**-0.123**	0.204	0.243	0.177
**TW**	-0.149	0.063	-0.183	-0.232	-0.012	0.352	-0.077	0.012	0.163	**0.335**	0.164	0.038
**PY**	-0.175	-0.238	-0.081	-0.273	0.056	0.281	0.33	0.118	0.085	0.373	**0.055**	-0.037
**ER**	0.101	0.052	0.119	-0.126	0.138	0.076	0.121	0.238	0.019	0.178	0.047	**0.043**

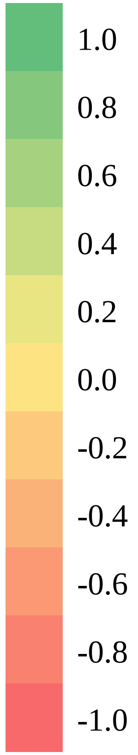

DF, days to 50% flowering; PH, plant height in cm; NP, number of panicle-bearing tillers; PL, length of panicle in cm; BM, biomass per plant in g; SF, spikelet fertility in %; YP, grain yield per plant in g; H%, hulling per cent; M%, milling percentage; TW, weight of 1000 grains in g; PY, Plot yield in kg per ha; ER, elongation ratio.

Supplementary Figure 1. The time curves for 32 NILs alongwith their parents for phenomics parameters measured at five stages

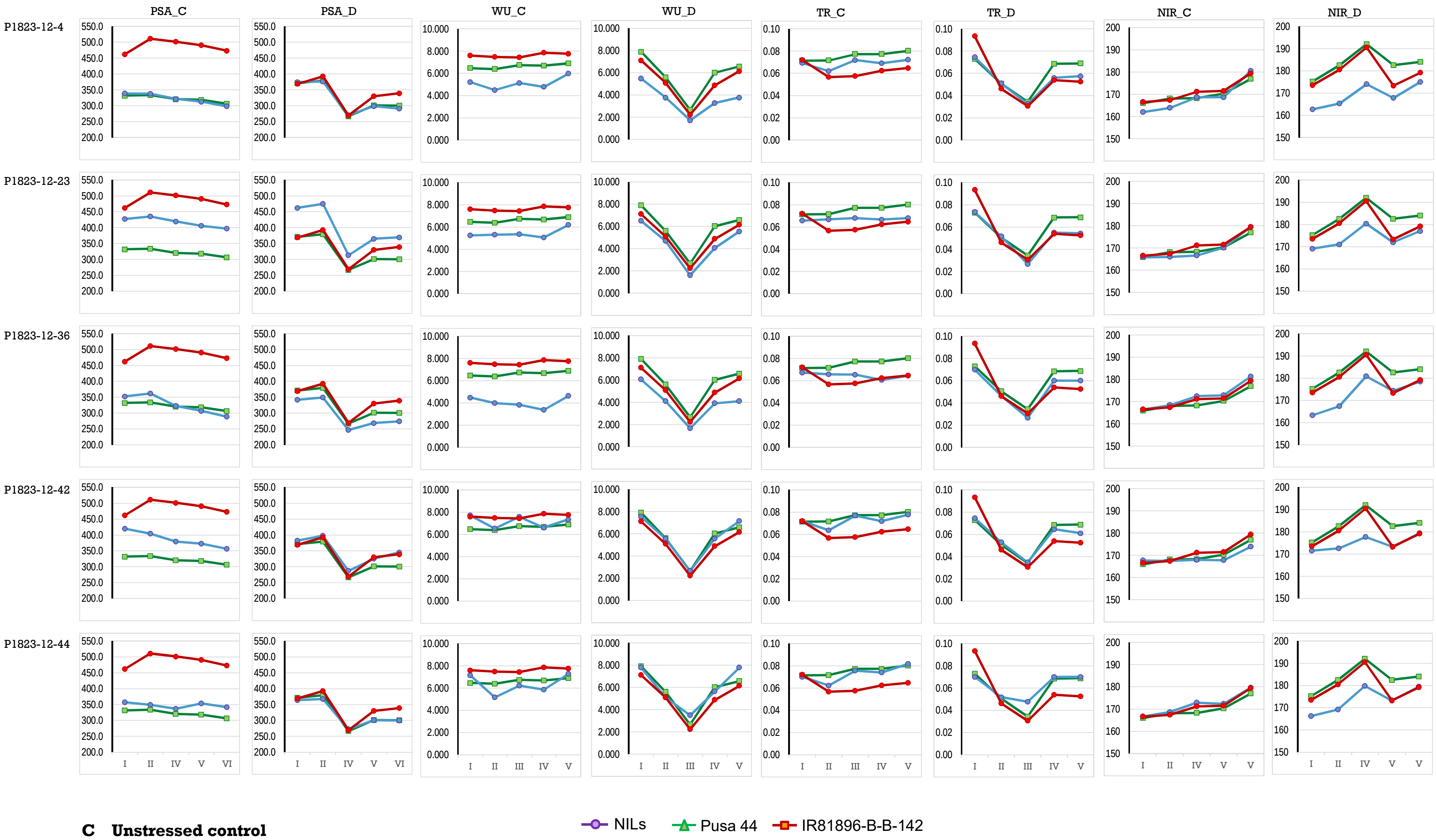

C Unstressed control

D Drought stressed

Supplementary Figure 1. Contd.

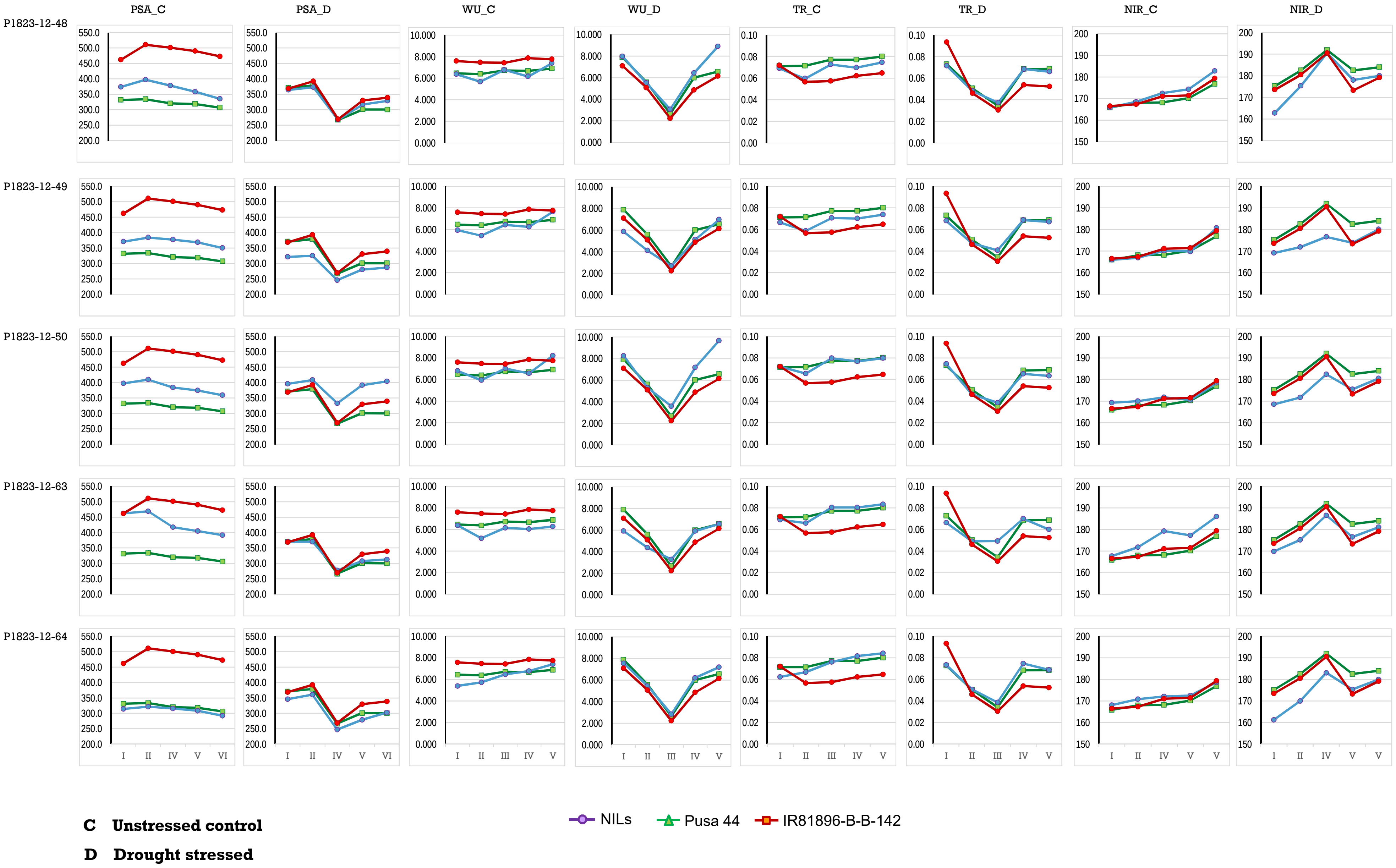

Supplementary Figure 1. Contd.

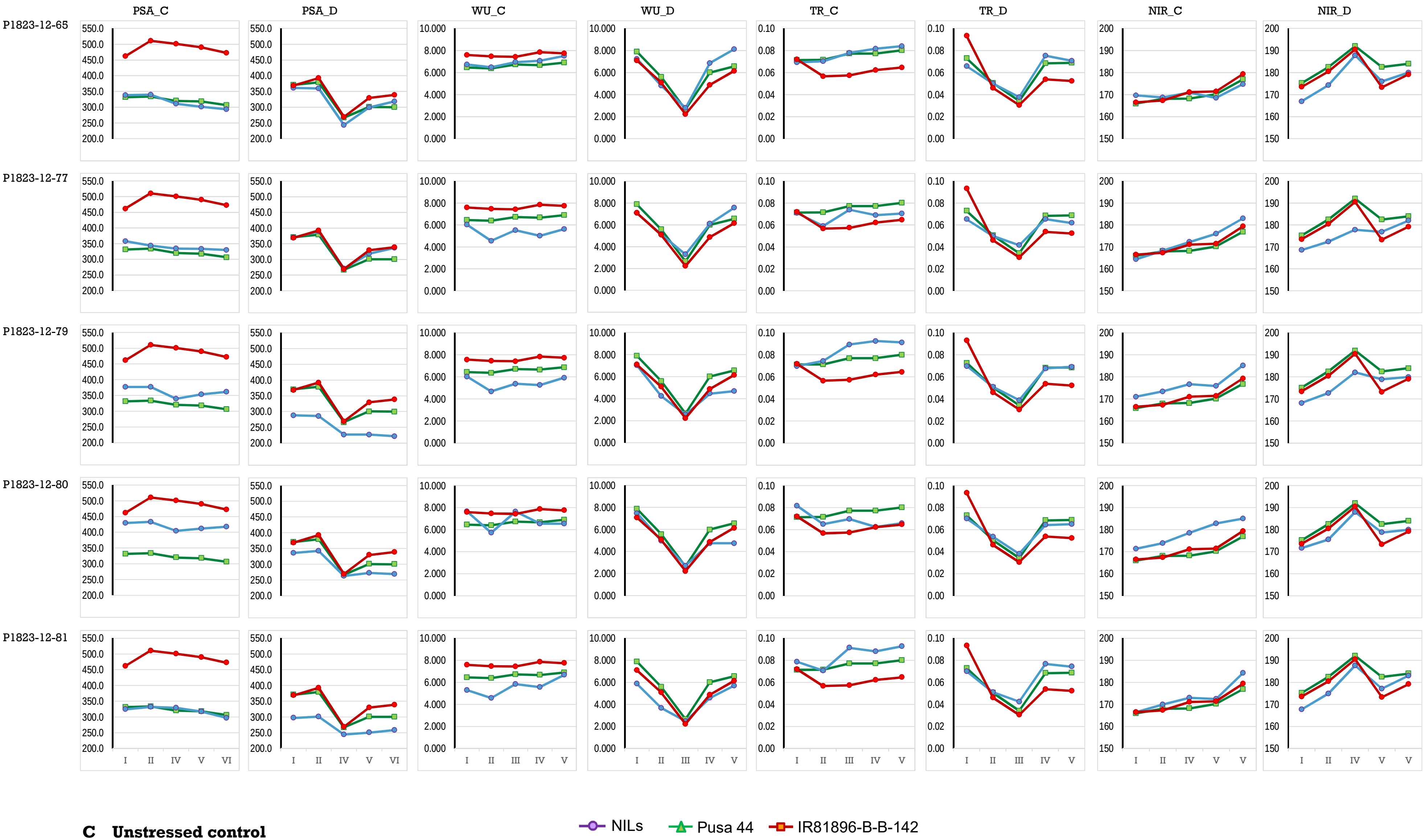

C **Unstressed control**

D **Drought stressed**

Supplementary Figure 1. Contd.

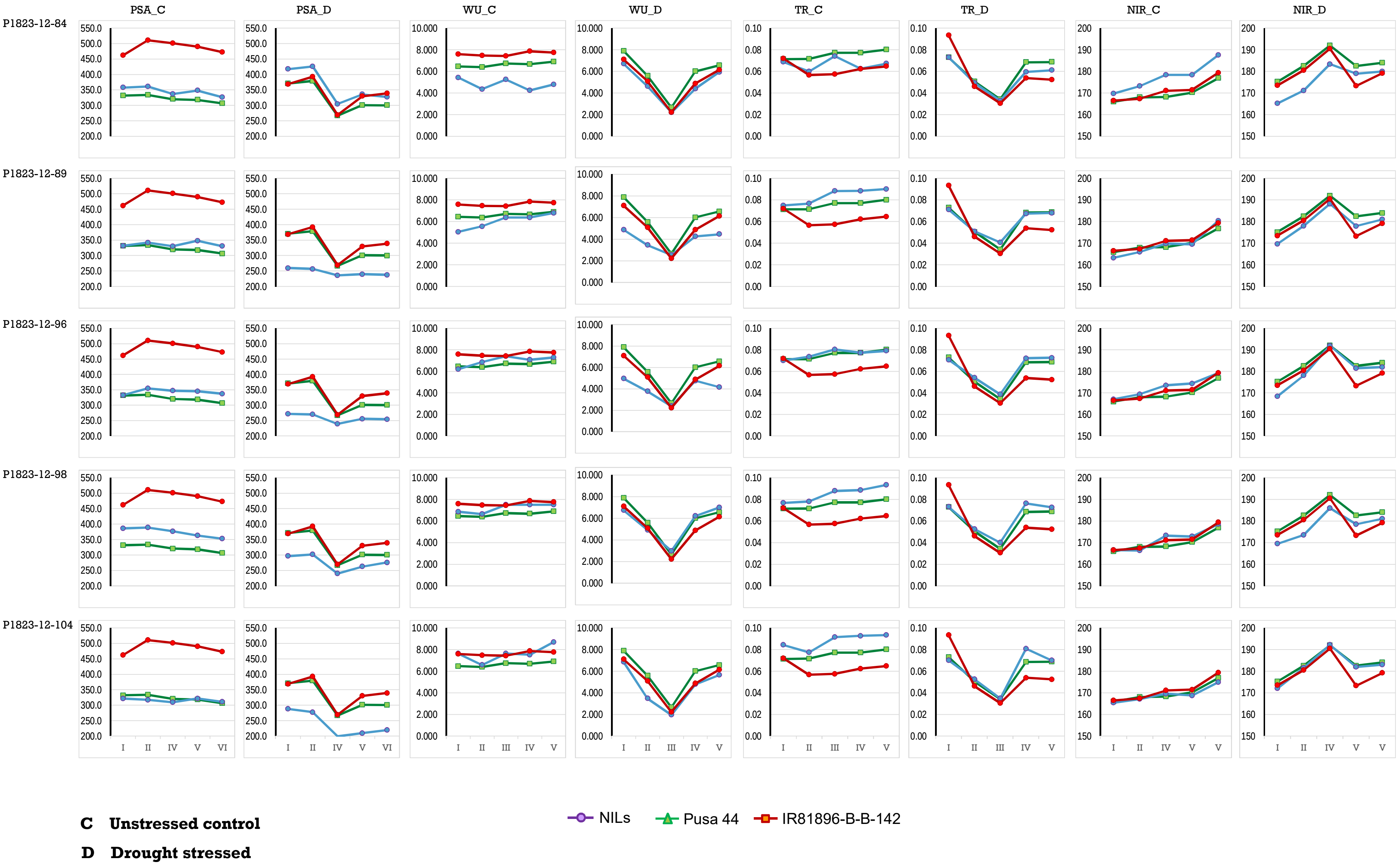

Supplementary Figure 1. Contd.

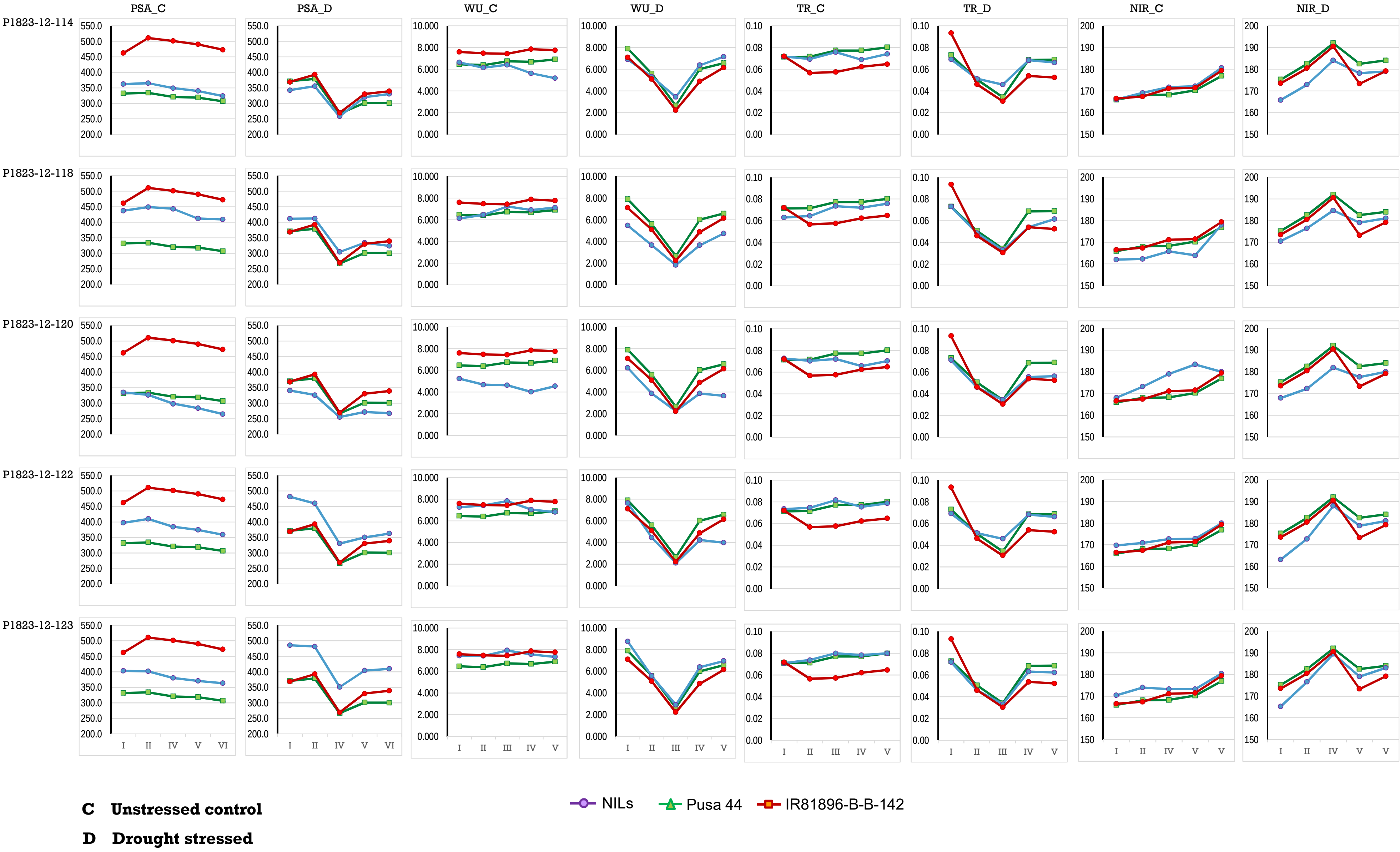

Supplementary Figure 1. Contd.

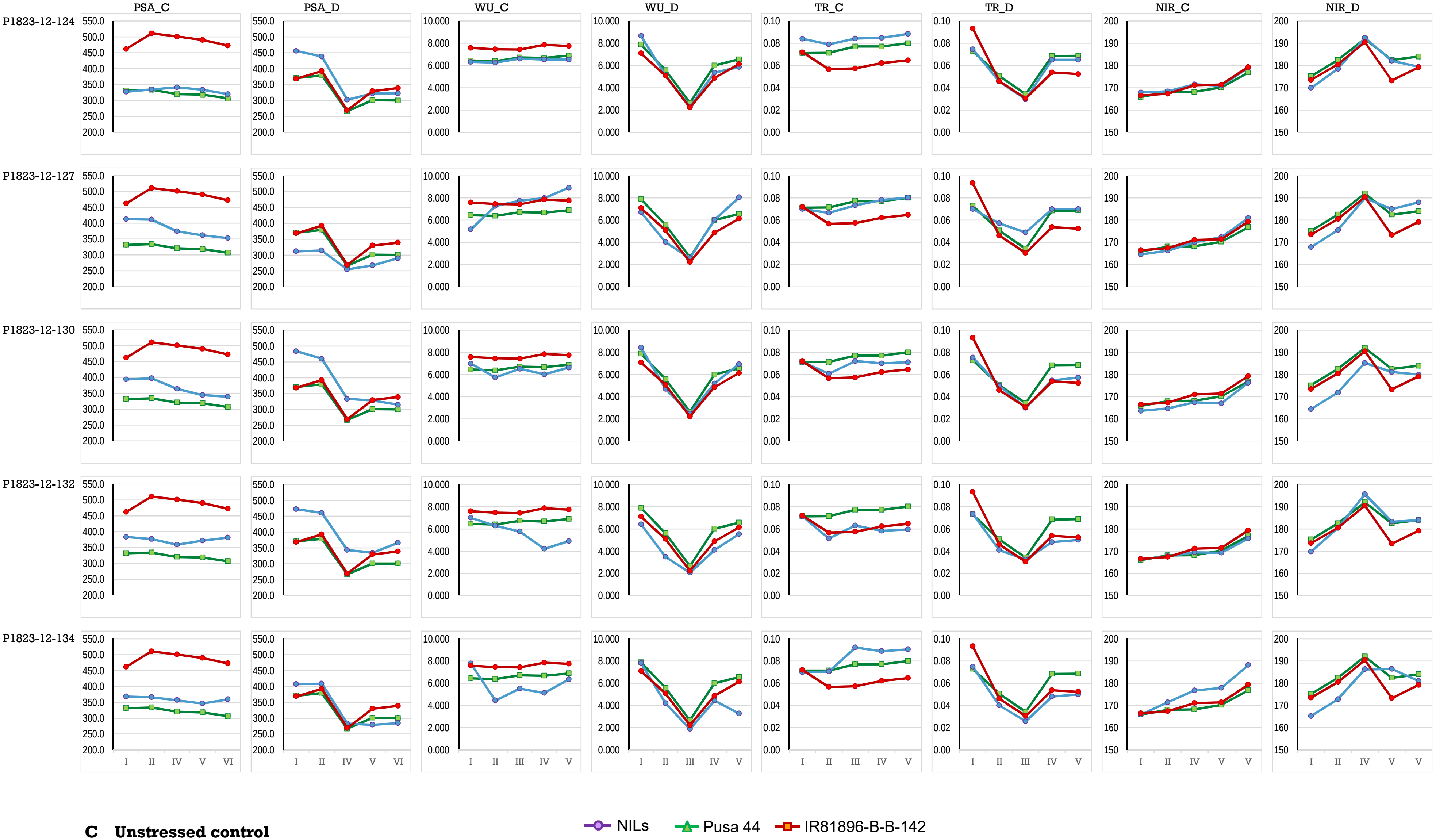

C Unstressed control

D Drought stressed

Supplementary Figure 1. Contd.

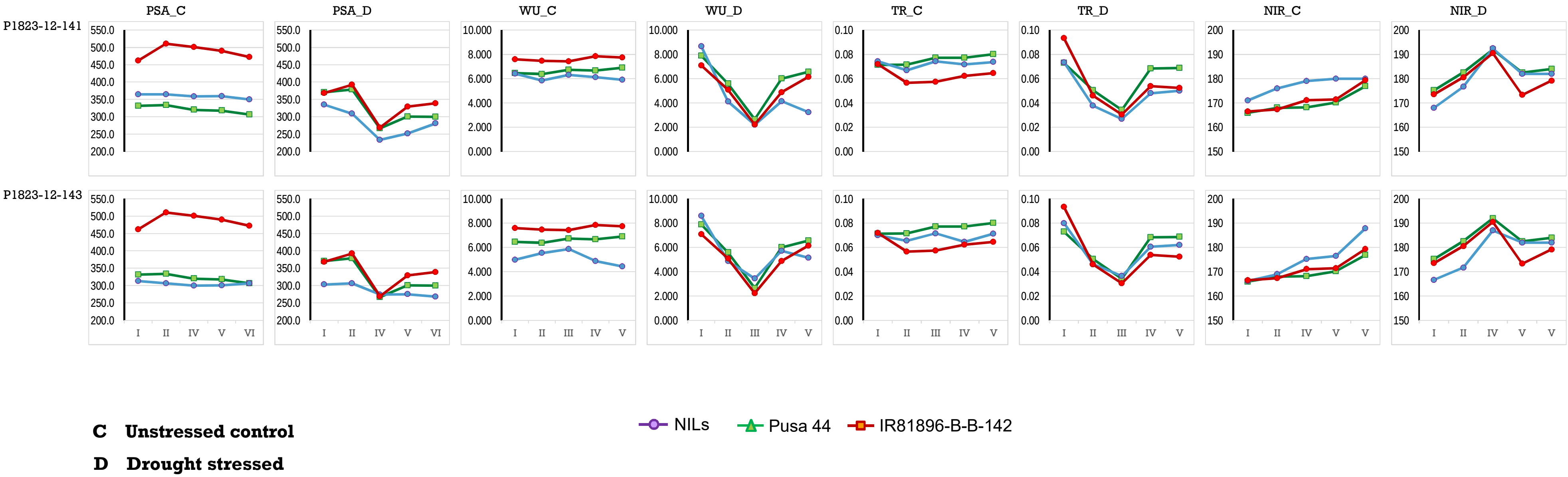

Supplement: Supplementary file 1 [file Data_Sheet_1.pdf]
